# Adaptive Image Processing: First Order PDE Constraint Regularizers and a Bilevel Training Scheme

**DOI:** 10.1007/s00332-023-09902-4

**Published:** 2023-03-03

**Authors:** Elisa Davoli, Irene Fonseca, Pan Liu

**Affiliations:** 1 Institute of Analysis and Scientific Computing, TU Wien, Wiedner Hauptstrasse 8-10, 1040 Vienna, Austria; 2 Department of Mathematics, Center of Nonlinear Analysis, Carnegie Mellon University, 5000 Forbes Avenue, Pittsburgh, PA 15213 USA; 3 Department of Radiology, First Medical Center of Chinese PLA General Hospital, Beijing, 100853 China

**Keywords:** Image processing, Optimal training scheme, First order differential operators, $\Gamma$-convergence, 26B30, 94A08, 47J20

## Abstract

A bilevel training scheme is used to introduce a novel class of regularizers, providing a unified approach to standard regularizers $TGV^2$ and $NsTGV^2$. Optimal parameters and regularizers are identified, and the existence of a solution for any given set of training imaging data is proved by $\Gamma$-convergence under a conditional uniform bound on the trace constant of the operators and a finite-null-space condition. Some first examples and numerical results are given.

## Introduction

Image processing aims at the reconstruction of an original “clean” image starting from a “distorted one”, namely from a datum which has been deteriorated or corrupted by noise effects or damaged digital transmission. The key idea of variational formulations in image-processing consists in rephrasing this problem as the minimization of an underlying functional of the form

$$\begin{aligned} {\mathcal {I}}(u):=\left\| u-u_\eta \right\| _{L^2(Q)}^2+{\mathcal {R}}_\alpha (u), \end{aligned}$$

where $u_\eta$ is a given corrupted image, $Q:=(-1/2,1/2)^N$ is the *N*-dimensional unit square (in image processing we usually take $N=2$, i.e., *Q* represents the domain of a square image) and ${\mathcal {R}}_\alpha$ is a regularizing functional, with $\alpha$ denoting the intensity parameter (which could be a positive scalar or a vector). Minimizing the functional ${\mathcal {I}}$ allows one to reconstruct a “clean” image based on the functional properties of the regularizer ${\mathcal {R}}_\alpha$.

Within the context of image denoising, for a fixed regularizer ${\mathcal {R}}_{\alpha }$ we seek to identify

$$\begin{aligned} u_{\alpha ,{\mathcal {R}}}:=\textrm{arg}\,\textrm{min}\left\{ \left\| u-u_\eta \right\| _{L^2(Q)}^2 +{\mathcal {R}}_\alpha (u):\,\, u\in L^2(Q)\right\} . \end{aligned}$$

An example is the ROF model (Rudin et al. 1992), in which the regularizer is taken to be ${\mathcal {R}}_\alpha (u):=\alpha TV(u)$, where *TV*(*u*) is the total variation of *u* [see, e.g. Ambrosio et al. (2000, Chapter 4)], $\alpha \in {\mathbb {R}}^+$ is the tuning parameter, and we have

1.1 $$\begin{aligned} u_{\alpha ,TV}:=\textrm{arg}\,\textrm{min}\left\{ \left\| u-u_\eta \right\| _{L^2(Q)}^2 +\alpha TV(u):\,\, u\in L^2(Q)\right\} . \end{aligned}$$

In view of the coercivity of the minimized functional, the natural class of competitors in (1.1) is *BV*(*Q*), the space of real-valued functions of bounded variation in *Q*. The trade-off between the denoising effects of the ROF-functional and its feature-preserving capabilities is encoded by the tuning parameter $\alpha \in {\mathbb {R}}^+$. Indeed, high values of $\alpha$ might lead to a strong penalization of the total variation of *u*, which in turn determines an over-smoothing effect and a resulting loss of information on the internal edges of the reconstructed image, while small values of $\alpha$ cause an unsatisfactory noise removal.

In order to determine the optimal $\alpha$, say ${\tilde{\alpha }}$, in De Los Reyes et al.  (2016, 2017) the authors proposed a bilevel training scheme, which was originally introduced in Machine Learning and later adopted by the imaging processing community (see Chen et al. 2013, 2014; Domke 2012; Tappen et al. 2007). The bilevel training scheme is a semi-supervised training scheme that optimally adapts itself to the given “clean data”. To be precise, let $(u_\eta ,u_c)$ be a pair of given images, where $u_\eta$ represents the corrupted version and $u_c$ stands for the original version, or the “clean” image. This training scheme searches for the optimal $\alpha$ so that the recovered image $u_{\alpha , TV}$, obtained in (1.1), minimizes the $L^2$-distance from the clean image ${u_c}$. An implementation of such training scheme, denoted by $({\mathcal {T}})$, equipped with total variation *TV* is 
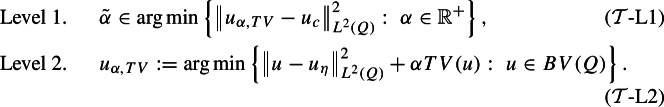


An important observation is that the geometric properties of the regularizer *TV* play an essential role in the identification of the reconstructed image $u_{\alpha , TV}$ and may lead to a loss of some fine texture in the image. The choice of a given regularizer ${\mathcal {R}}_{\alpha }$ is indeed a crucial step in the formulation of the denoising problem: on the one hand, the structure of the regularizer must be such that the removal of undesired noise effects is guaranteed, and on the other hand the disruption of essential details of the image must be prevented. For these reasons, various choices of regularizers have been proposed in the literature. For example, the second order total generalized variation, $TGV^2_\alpha$, defined as

1.2 $$\begin{aligned} TGV^2_{\alpha }(u)&:=\inf \left\{ \alpha _0\left|D u-v\right|_{{\mathcal {M}_b}(Q;{{{\mathbb {R}}}^N})} +\alpha _1\left|(\textrm{sym}\nabla ) v\right|_{{\mathcal {M}_b}(Q;\,{{\mathbb {R}}^{N\times N}})}:\right. \nonumber \\&\qquad \qquad \quad \left. v\in L^1(Q;{{{\mathbb {R}}}^N}),\,(\textrm{sym}\nabla ) v \in {\mathcal {M}_b}(Q;{{\mathbb {R}}^{N\times N}})\right\} , \end{aligned}$$

has been characterized in Bredies et al. (2010), where *Du* denotes the distributional gradient of *u*, $(\textrm{sym}\nabla ) v:=(\nabla v+\nabla ^Tv)/2$, ${\mathcal {M}_b}(Q;{{\mathbb {R}}^{N\times N}})$ is the space of bounded Radon measures in *Q* with values in ${{\mathbb {R}}^{N\times N}}$, $\alpha _0$ and $\alpha _1$ are positive tuning parameters, and $\alpha :=(\alpha _0,\alpha _1)$. A further possible choice for the regularizer is the non-symmetric counterpart of the $TGV^2_{\alpha }$-seminorm defined above, namely the $NsTGV^2_{\alpha }$ functional (see e.g., Valkonen et al. 2013; Valkonen 2017). The different regularizers have been shown to have several perks and drawbacks for image reconstruction. An important question is thus how to identify the regularizer that might provide the best possible image denoising for a given class of corrupted images.

To address this problem, it is natural to use a straightforward modification of scheme $({\mathcal {T}})$ by inserting different regularizers inside the training level 2 in (${\mathcal {T}}$-L2). For example, one could set

1.3 $$\begin{aligned}&\text {Level 1.} \qquad (\tilde{{\mathcal {R}}}_\alpha ):=\textrm{arg}\,\textrm{min}\left\{ \left\| u_{\alpha ,{\mathcal {R}}}-u_c\right\| _{L^2(Q)}^2:\,\,{\mathcal {R}}_\alpha \in \left\{ \alpha TV,TGV^2_{\alpha }, NsTGV_\alpha ^2 \right\} \right\} ,\nonumber \\&\text {Level 2.} \qquad u_{\alpha ,{\mathcal {R}}}:=\textrm{arg}\,\textrm{min}\left\{ \left\| u-u_\eta \right\| _{L^2(Q)}^2+{\mathcal {R}}_\alpha (u):\,\,u\in L^1(Q)\right\} . \end{aligned}$$

However, the finite number of possible choices for the regularizer within this training scheme would imply that the optimal regularizer $\tilde{{\mathcal {R}}}_{\alpha }$ would simply be determined by performing scheme $({\mathcal {T}})$ finitely many times, at each time with a different regularizer ${\mathcal {R}}_{\alpha }$. In turn, some possible texture effects for which an “intermediate” (or interpolated) reconstruction between the one provided by, say, $TGV^2_{\alpha }$ and $NsTGV^{2}_{\alpha }$, might be more accurate, would then be neglected in the optimization procedure. Therefore, one main challenge in the setup of such a training scheme is to give a meaningful interpolation between the regularizers used in (1.3), and also to guarantee that the collection of the corresponding functional spaces exhibits compactness and lower semicontinuity properties.

The aim of this paper is threefold. First, we propose a novel class of image-processing operators, the PDE-constrained total generalized variation operators, or $PGV^2_{\alpha ,\mathscr {B}}$, defined as

1.4 $$\begin{aligned} PGV^2_{\alpha ,{\mathscr {B}}}(u)&:=\inf \left\{ \alpha _0\left|D u-v\right|_{{\mathcal {M}_b}(Q;{{{\mathbb {R}}}^N})} +\alpha _1\left|{\mathscr {B}}v\right|_{{\mathcal {M}_b}(Q;\,{{\mathbb {R}}^{N\times N}})}:\right. \nonumber \\&\quad \qquad \qquad \left. v\in L^1(Q;{{{\mathbb {R}}}^N}),\,\mathscr {B} v\in {\mathcal {M}_b}(Q;{{\mathbb {R}}^{N\times N}})\right\} , \end{aligned}$$

for each $u\in L^1(Q;{{{\mathbb {R}}}^N})$, where ${\mathscr {B}}$ is a linear differential operator (see Sect. 2 and Definition 3.5) and $\alpha :=(\alpha _0,\alpha _1)$, with $\alpha _0,\,\alpha _1\in (0,+\infty )$. We also define the space of functions with bounded second order $PGV^2_{\alpha ,{\mathscr {B}}}$-seminorms

$$\begin{aligned} BPGV^2_{\alpha ,{\mathscr {B}}}(Q):=\left\{ u\in L^1(Q): \,\,{PGV_{\alpha ,{\mathscr {B}}}^2(u)<+\infty }\right\} . \end{aligned}$$

Note that if ${\mathscr {B}}:=\textrm{sym }\nabla$, then the operator $PGV^2_{\alpha ,{\mathscr {B}}}$ defined in (1.4) coincides with the operator $TGV^2_\alpha$ mentioned in (1.2). In fact, we will show that, under appropriate assumptions (see Definition 6.1), the class described in (1.4) provides a unified approach to some of the standard regularizers mentioned in (1.3), generalizing the results in Brinkmann et al. (2019) (see Sect. 7.2). Moreover, the collection of functionals described in (1.4) naturally incorporates the recent PDE-based approach to image denoising formulated in Barbu and Marinoschi (2017) via nonconvex optimal control problem, thus offering a very general and abstract framework to simultaneously describe a variety of different image-processing techniques. Adding to the model higher-order regularizations which can be different from the symmetric gradient additionally allows to enhance image reconstruction in one direction more than in the others, thus paving the way for furthering the study of anisotropic noise-reduction.

The second main goal of this article is the study of a training scheme optimizing the trade-off between effective reconstruction and fine image-detail preservation. That is, we propose a new bilevel training scheme that simultaneously yields the optimal regularizer $PGV^2_{\alpha ,{\mathscr {B}}}(u)$ in the class described in (1.4) and an optimal tuning parameter $\alpha$, so that the corresponding reconstructed image $u_{\alpha ,\mathscr {B}}$, obtained in Level 2 of the $({\mathcal {T}}^2_\theta )$-scheme (see (${\mathcal {T}}^2_\theta$-L2) below), minimizes the $L^2$-distance from the original clean image $u_c$. To be precise, in Sects. 3, 4, and 5 we study the improved training scheme ${\mathcal {T}}^2_\theta$for $\theta \in (0,1)$, defined as follows 
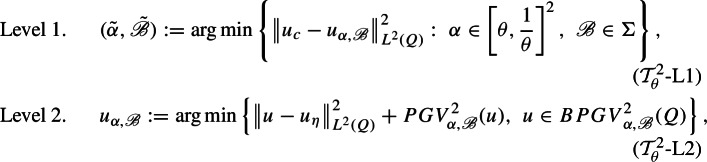
 where $\Sigma$ is an infinite collection of first order linear differential operators ${\mathscr {B}}$ (see Definitions 3.4, 5.1). We prove the existence of optimal solutions to (${\mathcal {T}}^2_\theta$-L1) by showing that the functional

1.5 $$\begin{aligned} {\mathcal {I}}_{\alpha ,{\mathscr {B}}}(u):=\left\| u-u_\eta \right\| _{L^2}^2+ {PGV^2_{\alpha ,{\mathscr {B}}}}(u) \end{aligned}$$

is continuous in the $L^1$ topology, in the sense of $\Gamma$-convergence, with respect to the parameters $\alpha$ and the operators ${\mathscr {B}}$ (see Theorem 4.2). A simplified statement of our main result (see Theorem 5.4) is the following.

### Theorem 1.1

Let $\theta \in (0,1)$. Then, the training scheme $({\mathcal {T}}^{2}_\theta )$admits at least one solution $({\tilde{\alpha }}, \tilde{{\mathscr {B}}})\in \left[ \theta ,\frac{1}{\theta }\right] ^{2}\times \Sigma$, and provides an associated optimally reconstructed image $u_{{\tilde{\alpha }},\tilde{{\mathscr {B}}}}\in BV(Q)$.

The collection $\Sigma$ of operators ${\mathscr {B}}$ used in (${\mathcal {T}}^2_\theta$-L1) has to satisfy several natural regularity and ellipticity assumptions, which are fulfilled by ${\mathscr {B}}:=\nabla$ and ${\mathscr {B}}:=\textrm{sym}\nabla$ (see Sect. 7.2.1). The general requirements on ${\mathscr {B}}$ that allow scheme $({\mathcal {T}}^2_\theta )$ to have a solution are listed on Assumptions 3.2 and 3.3. Later in Sect. 6, as the third main contribution of this article, we provide in Definition 6.1 a collection of operators ${\mathscr {B}}$ satisfying Assumptions 3.2 and 3.3 under some uniform bounds on the behavior of their traces and under finiteness of their null spaces. A simplified statement of our result is the following (see Theorem 6.5 for the detailed formulation).

### Theorem 1.2

Let $\mathscr {B}$ be a first order differential operator such that there exists a differential operator $\mathscr {A}$ for which $(\mathscr {A},\mathscr {B})$ is a training operator pair, namely $\mathscr {A}$ admits a fundamental solution having suitable regularity assumptions, and the pair $(\mathscr {A},\mathscr {B})$ fulfills a suitable integration-by-parts formula (see Definition 6.1 for the precise conditions). Then $\mathscr {B}$ is such that the training scheme $({\mathcal {T}}^2_\theta )$ admits a solution.

The requirements collected in Definition 6.1 and the analysis in Sect. 6 move from the observation that a fundamental property that the admissible operators ${\mathscr {B}}$ must satisfy is to ensure that the set of maps $v\in L^1(Q;{\mathbb {R}}^N)$ such that ${\mathscr {B}}v$ is a bounded Radon measure (henceforth denoted by $BV_{\mathscr {B}}(Q;{\mathbb {R}}^N)$) must embed compactly in $L^1(Q;{\mathbb {R}}^N)$. In the case in which ${\mathscr {B}}$ coincides with $\nabla$ or $\textrm{sym}\nabla$, a crucial ingredient is Kolmogorov–Riesz compactness theorem (see Brezis 2011, Theorem 4.26 and Proposition 6.6). In particular, for ${\mathscr {B}}=\textrm{sym}\nabla$ the key point of the proof is to guarantee that bounded sets ${\mathcal {F}}\subset BD(Q)$ satisfy

$$\begin{aligned} \lim _{\left|h\right|\rightarrow 0}\left\| \tau _h f-f\right\| _{L^1({{{\mathbb {R}}}^N})}=0 \text { uniformly in }{\mathcal {F}}, \end{aligned}$$

where $\tau _h f(\cdot ):=f(\cdot -h)$. This in turn relies on the formal computation

$$\begin{aligned} \tau _h f -f&= \delta _h*f-f=\delta _h*(\delta *f) -(\delta *f)\\&=\delta _h*\left( (\textrm{curlcurl} \phi ) *f\right) -(\textrm{curlcurl} \phi )*f\\&=\textrm{curlcurl} \left( (\delta _h*\phi -\phi )*f\right) , \end{aligned}$$

where $\phi$ is a fundamental solution for $\textrm{curlcurl}$, and where $\delta$ and $\delta _h$ denote the Dirac deltas centered in the origin and in *h*, respectively. In the case in which ${\mathscr {B}}=\textrm{sym}\nabla$ the conclusion then follows from the fact that one can perform an “integration by parts” in the right-hand side of the above formula, and estimate the quantity $\textrm{curlcurl}\left( (\delta _h*\phi -\phi )*f\right)$ by means of the total variation of $(\textrm{sym}\nabla )f$ and owing to the regularity of the fundamental solution of $\textrm{curlcurl}$. The operator $\mathscr {A}$ in Theorem 1.2 plays the role of $\textrm{curlcurl}$ in the case in which $\textrm{sym}\nabla$ is replaced by a generic operator ${\mathscr {B}}$. Definition 6.1 is given in such a way as to guarantee that the above formal argument is rigorously justified for a pair of operators $(\mathscr {A},{\mathscr {B}})$.

Finally, in Sect. 7.2 we give some explicit examples to show that our class of regularizers $PGV^2_{\alpha ,{\mathscr {B}}}$ includes the seminorms $TGV^2_{\alpha }$ and $NsTGV^2_{\alpha }$, as well as smooth interpolations between them.

We remark that the task of determining not only the optimal tuning parameter but also the optimal regularizer for given training image data $(u_\eta ,u_c)$, has been undertaken in Davoli and Liu (2018) where we have introduced one dimensional real order $TGV^r$ regularizers, $r\in [1,+\infty )$, as well as a bilevel training scheme that simultaneously provides the optimal intensity parameters and order of derivation for one-dimensional signals.

Our analysis is complemented by the very first numerical simulations of the proposed bilevel training scheme. Although this work focuses mainly on the theoretical analysis of the operators ${PGV^2_{\alpha ,{\mathscr {B}}}}$ and on showing the existence of optimal results for the training scheme $({\mathcal {T}}^2_\theta )$, in Sect. 7.3 a primal-dual algorithm for solving (${\mathcal {T}}^2_\theta$-L2) is discussed, and some preliminary numerical examples, such as image denoising, are provided.

With this article we initiate our study of the combination of PDE-constraints and bilevel training schemes in image processing. Future goals will be:

- the construction of a finite grid approximation in which the optimal result $(\tilde{\alpha },\tilde{{\mathscr {B}}})$ for the training scheme $({\mathcal {T}}^2_\theta )$can be efficiently determined, with an estimation of the approximation accuracy;
- spatially dependent differential operators and multi-layer training schemes. This will allow to specialize the regularization according to the position in the image, providing a more accurate analysis of complex textures and of images alternating areas with finer details with parts having sharpest contours (see also Fonseca and Liu 2017).

This paper is organized as follows: in Sect. 2 we collect some notations and preliminary results. In Sect. 3 we analyze the main properties of the $PGV^2_{\alpha ,{\mathscr {B}}}$-seminorms. The $\Gamma$-convergence result and the bilevel training scheme are the subjects of Sects. 4 and 5, respectively. We point out that the results in Sects. 3 and 4 are direct generalizations of the works in Bredies and Valkonen (2011), Bredies and Holler (1993). The novelty of our approach consists in providing a slightly stronger analysis of the behavior of the functionals in (1.5) by showing not only convergence of minimizers under convergence of parameters and regularizers, but exhibiting also a complete $\Gamma$-convergence result.

The expert Reader might skip Sects. 3–5, and proceed directly with the content of Sect. 6. Section 6 is devoted to the analysis of the space $BV_{\mathscr {B}}$ for suitable differential operators ${\mathscr {B}}$. The numerical implementation of some explicit examples is performed in Sect. 7.3.

## Notations and Preliminary Results

We collect below some notation that will be adopted in connection with differential operators. Let $N\in {\mathbb {N}}$ be given, and let $Q:=(-1/2,1/2)^N$ be the unit open cube in ${{{\mathbb {R}}}^N}$ centered in the origin and with sides parallel to the coordinate axes. ${\mathbb {M}}^{N^3}$ is the space of real tensors of order $N\times N\times N$. Also, ${\mathcal {D}}'(Q, {\mathbb {R}}^{N})$ and ${\mathcal {D}}'(Q, {\mathbb {R}}^{N\times N})$ stand for the spaces of distributions with values in ${\mathbb {R}}^{N}$ and ${\mathbb {R}}^{N\times N}$, respectively, and ${\mathbb {R}}^N_+$ denotes the set of vectors in ${\mathbb {R}}^N$ having positive entries.

For every open set $U\subset {\mathbb {R}}^N$, the notation ${\mathscr {B}}$ will be used for first order differential operators ${\mathscr {B}}:{\mathcal {D}}'(U;{\mathbb {R}}^N)\rightarrow {\mathcal {D}}'(U;{\mathbb {R}}^{N\times N})$ defined as

2.1 $$\begin{aligned} (\mathscr {B} v)_{lj}:=\sum _{i,k=1}^N B^i_{ljk} \frac{\partial }{\partial x_i} v_k \quad \text {for every }v\in {\mathcal {D}}'(U;{{{\mathbb {R}}}^N}),\quad l,j=1,\dots ,N, \end{aligned}$$

where $\frac{\partial }{\partial x_i}$ denotes the distributional derivative with respect to the *i*-th variable, and where $B^i\in {\mathbb {M}}^{N^3}$ for each $i=1,\dots ,N$.

Given a sequence $\left\{ {{\mathscr {B}}_n}\right\} _{n=1}^\infty$ of first order differential operators and a first order differential operator ${\mathscr {B}}$, with coefficients $\left\{ {B^i_n}\right\} _{n=1}^\infty$ and $B^i$, $i=1,\dots , N$, respectively, we say that ${\mathscr {B}}_n\rightarrow {\mathscr {B}}$ in $\ell ^{\infty }$ if

2.2 $$\begin{aligned} \left\| {\mathscr {B}}_n-{\mathscr {B}}\right\| _{\ell ^{\infty }}:=\sum _{i=1}^N\left\| B^i_n-B^i\right\| \rightarrow 0, \end{aligned}$$

where for $B\in {{\mathbb {M}}}^{N^3}$, $\Vert B\Vert$ stands for its Euclidean norm.

## The Space of Functions with Bounded *PGV*-Seminorm

### The Space $BV_{{\mathscr {B}}}$ and the Class of Admissible Operators

We generalize the standard total variation seminorm by using first order differential operators ${\mathscr {B}}$: ${\mathcal {D}}'(Q;{\mathbb {R}}^N) \rightarrow {\mathcal {D}}'(Q;{\mathbb {R}}^{N\times N} )$ in the form (2.1).

#### Definition 3.1

We define the space of tensor-valued functions $BV_{\mathscr {B}}(Q;{\mathbb {R}}^N)$ as

3.1 $$\begin{aligned} BV_{\mathscr {B}}(Q;{\mathbb {R}}^N ):=\left\{ u\in L^1 (Q; {\mathbb {R}}^N ):\,\, {\mathscr {B}}u\in {\mathcal {M}}_b (Q,{\mathbb {R}}^{N\times N} )\right\} , \end{aligned}$$

and we equip it with the norm

3.2 $$\begin{aligned} \left\| u\right\| _{BV_{\mathscr {B}}(Q;{\mathbb {R}}^N )}:=\left\| u\right\| _{L^1(Q;{\mathbb {R}}^N )} +\left|{\mathscr {B}}u\right|_{{\mathcal {M}}_b(Q;{\mathbb {R}}^{N\times N} )}. \end{aligned}$$

We refer to Raiţă (2019), Raiţă and Skorobogatova (2020) for some recents results on $BV_{\mathscr {B}}$-spaces for elliptic and cancelling operators, as well as to Kristensen and Raiţă (2022) for a study of associated Young measures. We point out that in the same way in which *BV* spaces relate to $W^{1,p}$-spaces, the spaces $BV_{\mathscr {B}}$ are connected to the theory of $W^{1,p}_{\mathscr {B}}$-spaces, cf. (Gmeineder and Raiţă 2019; Gmeineder et al. 2019; Raiţă 2018, 2019). See also Guerra and Raiţă (2020) for a related compensated-compactness study.

In order to introduce the class of admissible operators, we first list some assumptions on the operator ${\mathscr {B}}$.

#### Assumption 3.2

1. The space $BV_{\mathscr {B}}(Q;{\mathbb {R}}^N )$ is a Banach space with respect to the norm defined in (3.1).
2. The space $C^\infty (\overline{Q},{\mathbb {R}}^N )$ is dense in $BV_{\mathscr {B}}(Q;{\mathbb {R}}^N )$ in the strict topology. In other words, for every $u\in BV_{\mathscr {B}}(Q;{\mathbb {R}}^N )$ there exists $\left\{ {u_n}\right\} _{n=1}^\infty \subset C^\infty (\bar{Q}; {\mathbb {R}}^N )$ such that
  $$\begin{aligned} u_n\rightarrow u\text { strongly in }L^1(Q; {\mathbb {R}}^N )\text { and } \left|{\mathscr {B}}u_n\right|_{{\mathcal {M}}_b(Q;{\mathbb {R}}^{N\times N} )} \rightarrow \left|{\mathscr {B}}u\right|_{{\mathcal {M}}_b(Q;{\mathbb {R}}^{N\times N} )}. \end{aligned}$$
3. (Compactness) The injection of $BV_{\mathscr {B}}(Q; {\mathbb {R}}^N )$ into $L^1(Q;{\mathbb {R}}^N )$ is compact.

We point out that all requirements above are satisfied for ${\mathscr {B}}:=\nabla$.

#### Assumption 3.3

The following compactness property applies to a collection of operators $\left\{ {{\mathscr {B}}_n}\right\} _{n=1}^\infty$. For $\left\{ {v_n,{\mathscr {B}}_n}\right\} _{n=1}^\infty$ such that ${\mathscr {B}}_n$ satisfies Assumption 3.2 for every $n\in {\mathbb {N}}$, and

$$\begin{aligned} \sup \left\{ \left\| {\mathscr {B}}_n\right\| _{\ell ^{\infty }}+\left\| v_n\right\| _{BV_{{\mathscr {B}}_n} (Q; {\mathbb {R}}^N )}:\,\, n\in {\mathbb {N}}\right\} <+\infty , \end{aligned}$$

we assume that there exist ${\mathscr {B}}$ and $v\in BV_{{\mathscr {B}}}(Q;{\mathbb {R}}^N)$ such that, up to a subsequence (not relabeled),

$$\begin{aligned} v_n\rightarrow v\text { strongly in }L^1(Q; {\mathbb {R}}^N ), \end{aligned}$$

and

$$\begin{aligned} {{\mathscr {B}}_nv_n}\mathrel {\mathop {\rightharpoonup }\limits ^{*}}{{\mathscr {B}}v}\ \text {weakly}^{*}\ \text {in } {\mathcal {M}_b}(Q;{\mathbb {R}}^{N\times N} ). \end{aligned}$$

#### Definition 3.4

We denote by $\Pi$ the collection of operators ${\mathscr {B}}$ defined in (2.1), with finite dimensional null-space ${\mathcal {N}}({\mathscr {B}})$, and satisfying Assumption 3.2.

In Sect. 6 we will exhibit a subclass of operators ${\mathscr {B}}\in \Pi$ additionally fulfilling the compactness and closure Assumption 3.3.

### The *PGV*-Total Generalized Variation

We introduce below the definition of the PDE-constrained total generalized variation seminorms.

#### Definition 3.5

Let $u\in L^1(Q)$ be given. For every $\alpha =(\alpha _0,\alpha _1)\in {\mathbb {R}}^{2}_+$ and ${\mathscr {B}}$: ${\mathcal {D}}'(Q;{{{\mathbb {R}}}^N})\rightarrow {\mathcal {D}}'(Q;{{\mathbb {R}}^{N\times N}})$, ${\mathscr {B}}\in \Pi$, we consider the seminorm

3.3 $$\begin{aligned}{} & {} {PGV^2_{\alpha ,{\mathscr {B}}}}(u) \nonumber \\{} & {} \quad :=\inf \left\{ \alpha _0\left|Du-v\right|_{{\mathcal {M}_b}(Q;{\mathbb {R}}^N)} +\alpha _1\left|{\mathscr {B}}v\right|_{{\mathcal {M}_b}(Q;\,{\mathbb {R}}^{N\times N})}: \,\,v\in BV_{\mathscr {B}}(Q;{\mathbb {R}}^N)\right\} ,\nonumber \\ \end{aligned}$$

where the space $BV_{\mathscr {B}}$ is introduced in Definition 3.1.

We note that for all $\alpha \in {\mathbb {R}}^{2}_+$, the seminorms $PGV^2_{\alpha ,{\mathscr {B}}}$ are topologically equivalent. With a slight abuse of notation, in what follows we will write $PGV^2_{{\mathscr {B}}}$ instead of $PGV^2_{\alpha ,{\mathscr {B}}}$ whenever the dependence of the seminorm on a specific multi-index $\alpha \in {\mathbb {R}}^2_+$ will not be relevant for the presentation of the results.

We introduce below the set of functions with bounded *PDE*-generalized variation-seminorms.

#### Definition 3.6

We define

$$\begin{aligned} BPGV^2_{{\mathscr {B}}}(Q):=\left\{ u\in L^1(Q):\,\, PGV^2_{1,{\mathscr {B}}}(u)<+\infty \right\} , \end{aligned}$$

and we write

$$\begin{aligned} \left\| u\right\| _{BPGV^2_{{\mathscr {B}}}(Q)}:=\left\| u\right\| _{L^1(Q)}+PGV^2_{1,{\mathscr {B}}}(u). \end{aligned}$$

We next show that the $PGV^{2}_{{\mathscr {B}}}$-seminorm is finite if and only if the *TV*-seminorm is. The next three propositions show some basic properties of the $PGV^2$ regularizers. The expert Reader might skip their proof and proceed directly to Sect. 4.

#### Proposition 3.7

Let $u\in L^1(Q)$ and recall $PGV_{{\mathscr {B}}}^{2}(u)$ from Definition 3.5. Then, $PGV_{{\mathscr {B}}}^{2}(u)<+\infty$ if and only if $u\in BV(Q)$.

#### Proof

We notice that by setting $v=0$ in (3.3), we have

3.4 $$\begin{aligned} PGV_{{\mathscr {B}}}^{2}(u)\le \left|Du\right|_{{\mathcal {M}_b}(Q;{\mathbb {R}}^N)} \end{aligned}$$

for every $u\in L^1(Q)$. Thus, if $u\in BV(Q)$ then $PGV_{{\mathscr {B}}}^{2}(u)<+\infty$.

Conversely, assume that $PGV_{{\mathscr {B}}}^{2}(u)<+\infty$. Then, there exists $\bar{v}\in BV_{{\mathscr {B}}}(Q)$ such that

$$\begin{aligned} {PGV_{{\mathscr {B}}}^{2}}(u)\ge \left|Du-\bar{v}\right|_{{\mathcal {M}_b}(Q;{\mathbb {R}}^N)} +\left|{\mathscr {B}}\bar{v}\right|_{{\mathcal {M}_b}(Q;{{\mathbb {R}}^{N\times N}})}-1. \end{aligned}$$

It suffices to observe that

$$\begin{aligned} \left|Du\right|_{{\mathcal {M}_b}(Q;{\mathbb {R}}^N)}\le & {} \left|Du-\bar{v}\right|_{{\mathcal {M}_b}(Q;{\mathbb {R}}^N)} +\Vert \bar{v}\Vert _{L^1(Q;{\mathbb {R}}^N)}\\\le & {} {PGV_{{\mathscr {B}}}^{2}}(u)+1+\Vert \bar{v} \Vert _{L^1(Q;{\mathbb {R}}^N)}<+\infty . \end{aligned}$$

$$\square $$

We prove that the infimum problem in the right-hand side of (3.3) has a solution.

#### Proposition 3.8

Let $u\in BV(Q)$ and let $\mathscr {B}$ satisfy Assumption 3.2. Then, for $\alpha \in {\mathbb {R}}^2_+$ there exists a function $v\in BV_{{\mathscr {B}}}(Q;{{{\mathbb {R}}}^N})$ attaining the infimum in (3.3).

#### Proof

Let $u\in BV(Q)$ and, without loss of generality, assume that $\alpha =(1,1)$. In view of Proposition 3.7 we have ${PGV_{{\mathscr {B}}}^{2}}(u)<+\infty$.

The existence of a minimizer $v\in L^1(Q;{\mathbb {R}}^N)$ with ${\mathscr {B}}v\in {\mathcal {M}_b}(Q;{{\mathbb {R}}^{N\times N}})$ follows from the Direct Method of the calculus of variations. Indeed, let $\left\{ {v_n}\right\} _{n=1}^\infty \subset BV_{{\mathscr {B}}}(Q;{{{\mathbb {R}}}^N})$ be such that

$$\begin{aligned} \left|Du-v_n\right|_{{\mathcal {M}_b}(Q;{\mathbb {R}}^N)}+\left|{\mathscr {B}}v_n\right|_{{\mathcal {M}_b}(\Omega ;{{\mathbb {R}}^{N\times N}})} \le {PGV_{{\mathscr {B}}}^{2}}(u)+1/n \end{aligned}$$

for every $n\in {\mathbb {N}}$. Then,

3.5 $$\begin{aligned}{} & {} \left\| v_n\right\| _{L^1(Q;{\mathbb {R}}^N)}\le \left|Du-v_n\right|_{{\mathcal {M}_b}(Q;{\mathbb {R}}^N)} +\left|Du\right|_{{\mathcal {M}_b}(Q;{\mathbb {R}}^N)} \nonumber \\{} & {} \quad \le {PGV_{{\mathscr {B}}}^{2}}(u)+\left|Du\right|_{{\mathcal {M}_b}(Q;{\mathbb {R}}^N)}+1/n, \end{aligned}$$

and

3.6 $$\begin{aligned} \left|{\mathscr {B}}v_n\right|_{{\mathcal {M}_b}(Q;{{\mathbb {R}}^{N\times N}})}\le {PGV_{{\mathscr {B}}}^{2}}(u)+1/n, \end{aligned}$$

for every $n\in {\mathbb {N}}$. In view of Assumption 3.2, and together with (3.5) and (3.6), we obtain a function $v\in L^1(Q;{\mathbb {R}}^N)$ with ${\mathscr {B}}v\in {\mathcal {M}_b}(Q;{{\mathbb {R}}^{N\times N}})$ such that, up to the extraction of a subsequence (not relabeled), there holds

$$\begin{aligned} v_n\rightarrow v\quad \text {strongly in }L^1(Q;{\mathbb {R}}^N), \end{aligned}$$

and

$$\begin{aligned} {\liminf _{n\rightarrow \infty }}\left|{\mathscr {B}}v_n\right|_{{\mathcal {M}}_b(Q;{{\mathbb {R}}^{N\times N}})} \ge \left|{\mathscr {B}}v\right|_{{\mathcal {M}}_b(Q;{{\mathbb {R}}^{N\times N}})}. \end{aligned}$$

The minimality of *v* follows by lower-semicontinuity. $\square$

We close this section by studying the asymptotic behavior of the $PGV^{2}_{{\mathscr {B}}}$ seminorms in terms of the operator ${\mathscr {B}}$ for subclasses of $\Pi$ satisfying Assumption 3.3.

#### Proposition 3.9

Let $u\in BV(Q)$. Let $\left\{ {{\mathscr {B}}_n}\right\} _{n=1}^\infty \subset \Pi$ and $\left\{ {\alpha _n}\right\} _{n=1}^\infty \subset {\mathbb {R}}^{2}_+$ be such that ${\mathscr {B}}_n\rightarrow {\mathscr {B}}$ in $\ell ^{\infty }$ and

3.7 $$\begin{aligned} \alpha _n\rightarrow \alpha \in {\mathbb {R}}^{2}_+. \end{aligned}$$

Assume that $\left\{ {{\mathscr {B}}_n}\right\} _{n=1}^\infty$ satisfies Assumption 3.3. Then

$$\begin{aligned} \lim _{n\rightarrow \infty }PGV_{\alpha _n,{\mathscr {B}}_n}^{2}(u)=PGV_{\alpha ,{\mathscr {B}}}^{2}(u). \end{aligned}$$

#### Proof

We first claim that

3.8 $$\begin{aligned} {\liminf _{n\rightarrow \infty }}PGV^2_{\alpha _n,{\mathscr {B}}_n}(u)\ge PGV^2_{\alpha ,{\mathscr {B}}}(u). \end{aligned}$$

Indeed, by Proposition 3.8 for each $n\in {\mathbb {N}}$ there exists $v_n\in BV_{{\mathscr {B}}_n}(Q;{{{\mathbb {R}}}^N})$ such that, setting $\alpha _n=(\alpha _n^0,\alpha _n^1)$,

$$\begin{aligned} PGV^2_{\alpha _n,{\mathscr {B}}_n}(u)=\alpha _n^0\left|Du-v_n\right|_{{\mathcal {M}_b}(Q;{\mathbb {R}}^N)} +\alpha _n^1\left|{\mathscr {B}}_n v_n\right|_{{\mathcal {M}_b}(Q;{{\mathbb {R}}^{N\times N}})}. \end{aligned}$$

From (3.4) and (3.7),we see that

$$\begin{aligned} \alpha _n^0\left|Du-v_n\right|_{{\mathcal {M}_b}(Q;{\mathbb {R}}^N)}+\alpha _n^1 \left|{\mathscr {B}}_n v_n\right|_{{\mathcal {M}_b}(Q;{{\mathbb {R}}^{N\times N}})}\le \alpha _n^0\left|Du\right|<+\infty , \end{aligned}$$

which from (3.7) implies that $\sup \{\left\| v_n\right\| _{BV_{{\mathscr {B}}_n}(Q;{\mathbb {R}}^N)}+\left\| {\mathscr {B}}_n\right\| _{\ell ^\infty }\}$ is finite. Therefore, by Assumption 3.3 there exist ${\mathscr {B}}$ and $v\in BV_{{\mathscr {B}}}(Q)$ such that $v_n\rightarrow v$ strongly in $L^1(Q;{{{\mathbb {R}}}^N})$ and

3.9 $$\begin{aligned} {\liminf _{n\rightarrow \infty }}\left|{\mathscr {B}}_n v_n\right|_{{\mathcal {M}}_b(Q;{{\mathbb {R}}^{N\times N}})} \ge \left|{\mathscr {B}}v\right|_{{\mathcal {M}}_b(Q;{{\mathbb {R}}^{N\times N}})}. \end{aligned}$$

Fix $\varepsilon >0$. By (3.7), for *n* big enough there holds $\alpha _n^0\ge (1-\varepsilon )\alpha _0$, and $\alpha _n^1\ge (1-\varepsilon )\alpha _1$. Thus, by (3.9) we have

$$\begin{aligned} {\liminf _{n\rightarrow \infty }}\, PGV^2_{\alpha _n, {\mathscr {B}}_n}(u)&={\liminf _{n\rightarrow \infty }}\left[ \alpha _n^0\left|Du-v_n\right|_{{\mathcal {M}_b}(Q;{\mathbb {R}}^N)} +\alpha _n^1\left|{\mathscr {B}}_n v_n\right|_{{\mathcal {M}_b}(Q;{{\mathbb {R}}^{N\times N}})}\right] \\&\ge (1-\varepsilon )\alpha _0{\liminf _{n\rightarrow \infty }}\left|Du-v_n\right|_{{\mathcal {M}_b}(Q;{\mathbb {R}}^N)} +(1-\varepsilon )\alpha _1\\&\quad {\liminf _{n\rightarrow \infty }}\left|{\mathscr {B}}_n v_n\right|_{{\mathcal {M}_b}(Q;{{\mathbb {R}}^{N\times N}})}\\&\ge (1-\varepsilon )\alpha _0\left|Du-v\right|_{{\mathcal {M}_b}(Q;{\mathbb {R}}^N)}+(1-\varepsilon ) \alpha _1\left|{\mathscr {B}}v\right|_{{\mathcal {M}_b}(Q;{{\mathbb {R}}^{N\times N}})}\\&\ge (1-\varepsilon )PGV_{\alpha ,{\mathscr {B}}}^2(u), \end{aligned}$$

where in the last inequality we used (3.3). The arbitrariness of $\varepsilon$ concludes the proof of (3.8).

We now claim that

3.10 $$\begin{aligned} {\limsup _{n\rightarrow \infty }}\,PGV^2_{\alpha _n, {\mathscr {B}}_n}(u)\le PGV^2_{\alpha ,{\mathscr {B}}}(u). \end{aligned}$$

By Proposition 3.8 there exists $v\in BV_{{\mathscr {B}}}(Q;{{{\mathbb {R}}}^N})$ such that

$$\begin{aligned} PGV^2_{\alpha ,{\mathscr {B}}}(u)=\alpha _0\left|Du-v\right|_{{\mathcal {M}_b}(Q;{\mathbb {R}}^N)} +\alpha _1\left|{\mathscr {B}}v\right|_{{\mathcal {M}_b}(Q;{{\mathbb {R}}^{N\times N}})}. \end{aligned}$$

In view of the density result in Assumption 3.2, Statement 2, we may assume that $v\in C^\infty (Q;{{{\mathbb {R}}}^N})$ and, for $\varepsilon >0$ small,

3.11 $$\begin{aligned} PGV^2_{\alpha ,{\mathscr {B}}}(u)\ge \alpha _0\left|Du-v\right|_{{\mathcal {M}_b}(\Omega ;{\mathbb {R}}^N)} +\alpha _1\left|{\mathscr {B}}v\right|_{{\mathcal {M}_b}(\Omega ;{{\mathbb {R}}^{N\times N}})} -\varepsilon . \end{aligned}$$

Since

$$\begin{aligned} PGV^2_{\alpha _n,{\mathscr {B}}_n}(u)\le \alpha _n^0 \left|Du-v\right|_{{\mathcal {M}_b}(Q;{\mathbb {R}}^N)}+\alpha _n^1\left|{\mathscr {B}}_n v\right|_{{\mathcal {M}_b}(Q;{{\mathbb {R}}^{N\times N}})}, \end{aligned}$$

we obtain

$$\begin{aligned} {\limsup _{n\rightarrow \infty }}\,PGV^2_{\alpha _n,{\mathscr {B}}_n}(u)&\le \alpha _0\left|Du-v\right|_{{\mathcal {M}_b}(Q;{\mathbb {R}}^N)}+{\limsup _{n\rightarrow \infty }}\, \alpha _n^1\left|{\mathscr {B}}_n v\right|_{{\mathcal {M}_b}(Q;{{\mathbb {R}}^{N\times N}})}\\&\le \alpha _0\left|Du-v\right|_{{\mathcal {M}_b}(Q;{\mathbb {R}}^N)} +\alpha _1\left|{\mathscr {B}}v\right|_{{\mathcal {M}_b}(Q;{{\mathbb {R}}^{N\times N}})}\\&\le PGV^2_{\alpha ,{\mathscr {B}}}(u)+\varepsilon , \end{aligned}$$

where in the last inequality we used (3.11). Claim (3.10) is now asserted by the arbitrariness of $\varepsilon >0$. $\square$

## $\Gamma$-Convergence of Functionals Defined by *PGV*-Total Generalized Variation Seminorms

In this section we prove a $\Gamma$-convergence result with respect to the operator ${\mathscr {B}}$. For $r>0$ we denote [see (2.2)]

4.1 $$\begin{aligned} ({\mathscr {B}})_r:=\left\{ {\mathscr {B}}'\in \Pi :\,\,\left\| {\mathscr {B}}'-{\mathscr {B}}\right\| _{\ell ^\infty }\le r\right\} . \end{aligned}$$

Throughout this section, let $u_{\eta }\in L^2(Q)$ be a given datum representing a corrupted image.

### Definition 4.1

Let ${\mathscr {B}}\in \Pi$, $\alpha \in {\mathbb {R}}^{2}_+$. We define the functional ${\mathcal {I}}_{\alpha ,{\mathscr {B}}}$:$L^1(Q)\rightarrow [0,+\infty ]$ as

$$\begin{aligned} {\mathcal {I}}_{\alpha ,{\mathscr {B}}}(u):= {\left\{ \begin{array}{ll} \left\| u-u_\eta \right\| _{L^2(Q)}^2+ PGV^2_{\alpha ,{\mathscr {B}}}(u)&{} \quad \text { if }u\in BV(Q),\\ +\infty &{} \quad \text { otherwise. } \end{array}\right. } \end{aligned}$$

The following theorem is the main result of this section.

### Theorem 4.2

Let $\left\{ {{\mathscr {B}}_n}\right\} _{n=1}^\infty \subset \Pi$ satisfy Assumption 3.3, and let $\left\{ {\alpha _n}\right\} _{n=1}^\infty \subset {\mathbb {R}}^{2}_+$be such that ${\mathscr {B}}_n\rightarrow {\mathscr {B}}$ in $\ell ^{\infty }$ and $\alpha _n\rightarrow \alpha \in {\mathbb {R}}^{2}_+$. Then the functionals ${\mathcal {I}}_{\alpha _n,{\mathscr {B}}_n}$ satisfy the following compactness properties:

(Compactness) Let $u_n\in BV(Q)$, $n\in {\mathbb {N}}$, be such that

$$\begin{aligned} \sup \left\{ {\mathcal {I}}_{\alpha _n,{\mathscr {B}}_n}(u_n):\,\,n\in {\mathbb {N}}\right\} <+\infty . \end{aligned}$$

Then there exists $u\in BV(Q)$ such that, up to the extraction of a subsequence (not relabeled),

$$\begin{aligned} u_n\mathrel {\mathop {\rightharpoonup }\limits ^{*}}u\text { weakly}^*\text { in }BV(Q). \end{aligned}$$

Additionally, ${\mathcal {I}}_{\alpha _n,{\mathscr {B}}_n}$ $\Gamma$-converges to ${\mathcal {I}}_{\alpha ,{\mathscr {B}}}$ in the $L^1$ topology. To be precise, for every $u\in BV(Q)$ the following two conditions hold:

(Liminf inequality) If

$$\begin{aligned} u_n\rightarrow u\text { in }L^1(Q) \end{aligned}$$

then

$$\begin{aligned} {\mathcal {I}}_{\alpha ,{\mathscr {B}}}(u)\le \liminf _{n\rightarrow +\infty } {\mathcal {I}}_{\alpha _n,{\mathscr {B}}_n}(u_n). \end{aligned}$$

(Recovery sequence) For each $u\in BV(Q)$, there exists $\left\{ {u_n}\right\} _{n=1}^\infty \subset BV(Q)$ such that

$$\begin{aligned} u_n\rightarrow u\text { in }L^1(Q) \end{aligned}$$

and

$$\begin{aligned} \limsup _{n\rightarrow +\infty }{\mathcal {I}}_{\alpha _n,{\mathscr {B}}_n}(u_n) \le {\mathcal {I}}_{\alpha ,{\mathscr {B}}}(u). \end{aligned}$$

We subdivide the proof of Theorem 4.2 into two propositions.

For ${\mathscr {B}}\in \Pi$, we consider the projection operator

$$\begin{aligned} {\mathbb {P}}_{\mathscr {B}}:L^1(Q;{{{\mathbb {R}}}^N})\rightarrow {\mathcal {N}}({\mathscr {B}}). \end{aligned}$$

Note that this projection operator is well defined owing to the assumption that ${\mathcal {N}}({\mathscr {B}})$ is finite dimensional [see Brezis (2011, p. 38, Definition and Example 2) and Breit et al. (2017, Subsection 3.1)].

Next we have an enhanced version of Korn’s inequality.

### Proposition 4.3

Let ${\mathscr {B}}\in \Pi$ and let $r>0$ be small enough so that elements of $({\mathscr {B}})_r$ have finite dimensional kernel. Then, under Assumption 3.3 there exists a constant $C=C({\mathscr {B}},Q{,r})$, depending only on ${\mathscr {B}}$, on the domain *Q*, and on *r*, such that

4.2 $$\begin{aligned} \left\| v-{\mathbb {P}}_{{\mathscr {B}}'}(v)\right\| _{L^1(Q;{{{\mathbb {R}}}^N})} \le C \left|{\mathscr {B}}' v\right|_{{\mathcal {M}}_b(Q;{{\mathbb {R}}^{N\times N}})}, \end{aligned}$$

for all $v\in L^1(Q)$ and ${\mathscr {B}}'\in ({\mathscr {B}})_r$.

### Proof

Suppose that (4.2) fails. Then there exist sequences $\left\{ {{\mathscr {B}}_n}\right\} _{n=1}^\infty \subset ({\mathscr {B}})_r$ and $\left\{ {v_n}\right\} _{n=1}^\infty \subset L^1(Q)$ such that

$$\begin{aligned} \left\| v_n-{\mathbb {P}}_{{\mathscr {B}}_n}(v_n)\right\| _{L^1(Q;{{{\mathbb {R}}}^N})} \ge n \left|{\mathscr {B}}_n v_n\right|_{{\mathcal {M}}_b(Q;{{\mathbb {R}}^{N\times N}})} \end{aligned}$$

for every $n\in {{\mathbb {N}}}$. Up to a normalization, we can assume that

4.3 $$\begin{aligned} \left\| v_n-{\mathbb {P}}_{{\mathscr {B}}_n}(v_n)\right\| _{L^1(Q;{{{\mathbb {R}}}^N})}=1 \text { and }\left|{\mathscr {B}}_n v_n\right|_{{\mathcal {M}}_b(Q;{{\mathbb {R}}^{N\times N}})}\le 1/n \end{aligned}$$

for every $n\in {{\mathbb {N}}}$. Since $\left\{ {{\mathscr {B}}_n}\right\} _{n=1}^\infty \subset ({\mathscr {B}})_r$, up to a subsequence (not relabeled), we have ${\mathscr {B}}_n\rightarrow \tilde{{\mathscr {B}}}$ in $\ell ^\infty$, for some $\tilde{{\mathscr {B}}}\in ({\mathscr {B}})_r$. Next, let

$$\begin{aligned} \tilde{v}_n:= v_n-{\mathbb {P}}_{{\mathscr {B}}_n}(v_n). \end{aligned}$$

Note that for each $n\in {\mathbb {N}}$

4.4 $$\begin{aligned} {\mathbb {P}}_{{\mathscr {B}}_n}(\tilde{v}_n)=0. \end{aligned}$$

Thus, by (4.3) we have

4.5 $$\begin{aligned} \left\| \tilde{v}_n\right\| _{L^1(Q;{{{\mathbb {R}}}^N})}=1\text { and } \left|{\mathscr {B}}_n\tilde{v}_n\right|_{{\mathcal {M}}_b(Q;{{\mathbb {R}}^{N\times N}})}\le 1/n. \end{aligned}$$

In view of Assumption 3.3, up to a further subsequence (not relabeled), there exists $\tilde{v}\in BV_{\tilde{{\mathscr {B}}}}(Q;{{{\mathbb {R}}}^N})$ such that $\tilde{v}_n\rightarrow \tilde{v}$ strongly in $L^1(Q)$ and $|\tilde{{\mathscr {B}}}\tilde{v}|_{{\mathcal {M}}_b(Q;{{\mathbb {R}}^{N\times N}})}=0$. Moreover, in view of (4.5), we also have $\left\| \tilde{v}\right\| _{L^1(Q;{{{\mathbb {R}}}^N})}=1$.

By the joint continuity of the projection operator, by (4.4) we have

$$\begin{aligned} \left\| {\mathbb {P}}_{\tilde{{\mathscr {B}}}}(\tilde{v})\right\| _{L^1(Q)} =\left\| {\mathbb {P}}_{{\mathscr {B}}_n}(\tilde{v}_n) -{\mathbb {P}}_{\tilde{{\mathscr {B}}}}(\tilde{v})\right\| _{L^1(Q)} \le C{\left\| {\mathscr {B}}_n-{\mathscr {B}}\right\| _{\ell ^\infty }+}\left\| \tilde{v} -\tilde{v}_n\right\| _{L^1(Q)} \rightarrow 0. \end{aligned}$$

Thus, ${\mathbb {P}}_{\tilde{{\mathscr {B}}}}(\tilde{v})=0$. However, $|\tilde{{\mathscr {B}}}\tilde{v}|_{{\mathcal {M}}_b(Q;{{\mathbb {R}}^{N\times N}})}=0$ implies that $\tilde{v}\in {\mathcal {N}}[\tilde{{\mathscr {B}}}]$ with $\tilde{v}=P_{\tilde{{\mathscr {B}}}}(\tilde{v})$, and hence we must have $\tilde{v}=0$, contradicting the fact that $\left\| \tilde{v}\right\| _{L^1(Q;{{{\mathbb {R}}}^N})}=1$. $\square$

The following proposition is instrumental for establishing the liminf inequality.

### Proposition 4.4

Let $\left\{ {{\mathscr {B}}_n}\right\} _{n=1}^\infty \subset \Pi$ satisfy Assumption 3.3, and let $\left\{ {\alpha _n}\right\} _{n=1}^\infty \subset {\mathbb {R}}^{2}_+$ be such that ${\mathscr {B}}_n\rightarrow {\mathscr {B}}$ in $\ell ^{\infty }$ for ${\mathscr {B}}\in \Pi$, and $\alpha _n\rightarrow \alpha \in {\mathbb {R}}^{2}_+$. For every $n\in {\mathbb {N}}$ let $u_n\in BV(Q)$ be such that

4.6 $$\begin{aligned} \sup \left\{ {\mathcal {I}}_{\alpha _n,{\mathscr {B}}_n}(u_n):\,\,n\in {\mathbb {N}}\right\} <+\infty . \end{aligned}$$

Then there exists $u\in BV(Q)$ such that, up to the extraction of a subsequence (not relabeled),

4.7 $$\begin{aligned} u_n\mathrel {\mathop {\rightharpoonup }\limits ^{*}}u\text { weakly}^*\text { in }BV(Q) \end{aligned}$$

and

$$\begin{aligned} \liminf _{n\rightarrow \infty }{PGV_{\alpha _n,{\mathscr {B}}_n}^{2}(u_n)} \ge {PGV^{2}_{\alpha ,{\mathscr {B}}}(u)}. \end{aligned}$$

### Proof

Fix $r>0$ and recall the definition of $({\mathscr {B}})_r$ from (4.1). We claim that if *r* is small enough then there exists $C_r>0$ such that

4.8 $$\begin{aligned} \left\| u\right\| _{BPGV^2_{{\mathscr {B}}'}(Q)}\le \left\| u\right\| _{BV(Q)} \le C_r \left\| u\right\| _{BPGV^2_{{\mathscr {B}}'}(Q)}, \end{aligned}$$

for all $u\in BV(Q)$ and ${\mathscr {B}}'\in ({\mathscr {B}})_r$.

Indeed, by Definitions 3.5 and 3.6 we always have

$$\begin{aligned} \left\| u\right\| _{BPGV^2_{{\mathscr {B}}'}(Q)}\le \left\| u\right\| _{BV(Q)}, \end{aligned}$$

for all ${\mathscr {B}}'\in \Pi$ and $u\in BV(Q)$.

The crucial step is to prove that the second inequality in (4.8) holds. Set

$$\begin{aligned} {\mathcal {N}}_r({\mathscr {B}}):=\{\omega \in L^1(Q;{\mathbb {R}}^N): \,\text { there exists }\,{\mathscr {B}}'\in ({\mathscr {B}})_r \,\text { for which }\,\omega \in {\mathcal {N}}({\mathscr {B}}')\}. \end{aligned}$$

We claim that there exists $C>0$, depending on *r*, such that for each $u\in BV(Q)$ and $\omega \in {\mathcal {N}}_r({\mathscr {B}})$ we have

4.9 $$\begin{aligned} \left|Du\right|_{{{\mathcal {M}_b}(Q;{{{\mathbb {R}}}^N})}}\le C\left( \left|Du-\omega \right|_{{{\mathcal {M}_b}(Q;{{{\mathbb {R}}}^N})}}+\left\| u\right\| _{L^1(Q)}\right) . \end{aligned}$$

Suppose that (4.9) fails. Then we find sequences $\left\{ {u_n}\right\} _{n=1}^\infty \subset BV(Q)$ and $\left\{ {\omega _n}\right\} _{n=1}^\infty \subset {\mathcal {N}}_r({\mathscr {B}})$ such that

$$\begin{aligned} \left|Du_n\right|_{{{\mathcal {M}_b}(Q;{{{\mathbb {R}}}^N})}}\ge n\left( \left|Du_n-\omega _n\right|_{{{\mathcal {M}_b}(Q;{{{\mathbb {R}}}^N})}} +\left\| u_n\right\| _{L^1(Q)}\right) \end{aligned}$$

for every $n\in {{\mathbb {N}}}$. Thus, up to a normalization, we can assume that

4.10 $$\begin{aligned} \left|Du_n\right|_{{\mathcal {M}_b}(Q;{{{\mathbb {R}}}^N})}=1 \end{aligned}$$

and

4.11 $$\begin{aligned} \left|Du_n-\omega _n\right|_{{\mathcal {M}_b}(Q;{{{\mathbb {R}}}^N})}+\left\| u_n\right\| _{L^1(Q)}\le 1/n, \end{aligned}$$

which implies that $u_n\rightarrow 0$ strongly in $L^1(Q)$ and

4.12 $$\begin{aligned} \left|Du_n-\omega _n\right|_{{\mathcal {M}_b}(Q;{{{\mathbb {R}}}^N})}\rightarrow 0. \end{aligned}$$

By (4.10) and (4.11), it follows that $\left|\omega _n\right|_{{\mathcal {M}_b}(Q;{{{\mathbb {R}}}^N})}$ is uniformly bounded, and hence, up to a subsequence (not relabeled), there exists $\omega \in {{\mathcal {M}_b}(Q;{{{\mathbb {R}}}^N})}$ such that $\omega _n\mathrel {\mathop {\rightharpoonup }\limits ^{*}}\omega$ in ${{\mathcal {M}_b}(Q;{{{\mathbb {R}}}^N})}$. For every $n\in {\mathbb {N}}$ let ${\mathscr {B}}_n'\in ({\mathscr {B}})_r$ be such that $\omega _n\in {\mathcal {N}}({\mathscr {B}}_n')$. Then ${\mathscr {B}}_n' \omega _n=0$ for all $n\in {\mathbb {N}}$. Since $\left\| {\mathscr {B}}_n'-{\mathscr {B}}\right\| _{\ell ^{\infty }}<r$, in particular the sequence $\left\{ {\omega _n,{\mathscr {B}}_n'}\right\} _{n=1}^\infty \subset L^1({{\mathcal {M}_b}(Q;{{{\mathbb {R}}}^N})})\times \Pi$ fulfills Assumption 3.3, and hence, upon extracting a further subsequence (not relabeled), there holds

$$\begin{aligned} \omega _n\rightarrow \omega _0\quad \text {strongly in }L^1(Q;{\mathbb {R}}^N). \end{aligned}$$

Additionally, since $u_n\rightarrow 0$ strongly in $L^1(Q)$, we infer that $Du_n\rightarrow 0$ in the sense of distributions. Therefore, by (4.12) we deduce that $\omega _0=0$. Using again (4.11), we conclude that

$$\begin{aligned} \left|Du_n\right|_{{\mathcal {M}}_b(Q;{{{\mathbb {R}}}^N})}\rightarrow 0, \end{aligned}$$

which contradicts (4.10). This completes the proof of (4.9).

We are now ready to prove the second inequality in (4.8), i.e.,

4.13 $$\begin{aligned} \left\| u\right\| _{BV(Q)}\le C_r \left\| u\right\| _{BPGV^2_{{\mathscr {B}}'}(Q)} \end{aligned}$$

for some constant $C_r>0$, and for all ${\mathscr {B}}'\in ({\mathscr {B}})_r$.

Fix ${\mathscr {B}}'\in ({\mathscr {B}})_r$, and by Proposition 3.8 let $v_{{\mathscr {B}}'}$ satisfy

4.14 $$\begin{aligned} PGV_{{\mathscr {B}}'}^2(u)=\left|Du-v_{{\mathscr {B}}'}\right|_{{\mathcal {M}_b}(Q;{\mathbb {R}}^N)} +\left|{\mathscr {B}}' v_{{\mathscr {B}}'}\right|_{{\mathcal {M}_b}(Q;{{\mathbb {R}}^{N\times N}})}. \end{aligned}$$

Since ${\mathbb {P}}_{{\mathscr {B}}'}[v_{{\mathscr {B}}'}]\in {\mathcal {N}}_r({\mathscr {B}})$, we have

$$\begin{aligned} \left|Du\right|_{{\mathcal {M}_b}(Q;{{{\mathbb {R}}}^N})}&\le C(\left|Du-{\mathbb {P}}_{{\mathscr {B}}'}[v_{{\mathscr {B}}'}]\right|_{{\mathcal {M}_b}(Q;{{{\mathbb {R}}}^N})}+\left\| u\right\| _{L^1(Q)})\\&\le C(\left|Du-v_{{\mathscr {B}}'}\right|_{{\mathcal {M}_b}(Q;{{{\mathbb {R}}}^N})}+\left|v_{{\mathscr {B}}'} -{\mathbb {P}}_{{\mathscr {B}}'}[v_{{\mathscr {B}}'}]\right|_{{\mathcal {M}_b}(Q;{{{\mathbb {R}}}^N})}+\left\| u\right\| _{L^1(Q)})\\&\le C(\left|Du-v_{{\mathscr {B}}'}\right|_{{\mathcal {M}_b}(Q;{{{\mathbb {R}}}^N})}+C'\left|{{\mathscr {B}}'} v_{{\mathscr {B}}'}\right|_{{\mathcal {M}_b}(Q;{{\mathbb {R}}^{N\times N}})}+\left\| u\right\| _{L^1(Q)})\\&\le (C+C')\left[ \left|Du-v_{{\mathscr {B}}'}\right|_{{\mathcal {M}_b}(Q;{{{\mathbb {R}}}^N})}+\left|{{\mathscr {B}}'} v_{{\mathscr {B}}'}\right|_{{\mathcal {M}_b}(Q;{{\mathbb {R}}^{N\times N}})}+\left\| u\right\| _{L^1(Q)}\right] \\&=(C+C') \left[ PGV_{{\mathscr {B}}'}^2(u)+ \left\| u\right\| _{L^1(Q)}\right] , \end{aligned}$$

where in the first inequality we used (4.9), the third inequality follows by (4.2), and in the last equality we invoked (4.14). Defining $C_r:=C+C'+1$, we obtain

$$\begin{aligned} \left\| u\right\| _{BV(Q)}=\left\| u\right\| _{L^1(Q)}+\left|Du\right|_{{\mathcal {M}}_b (Q;{{{\mathbb {R}}}^N})}\le C_r (PGV_{{\mathscr {B}}'}^2(u)+ \left\| u\right\| _{L^1(Q)}) =C_r \left\| u\right\| _{BPGV_{{\mathscr {B}}'}^2(Q)} \end{aligned}$$

and we conclude (4.13).

Now we prove the compactness property. Fix $\varepsilon >0$. We first observe that, since $\alpha _n\rightarrow \alpha \in {{\mathbb {R}}}^2_+$, for $\alpha _n=(\alpha _n^0,\alpha _n^1)$, and for *n* small enough there holds

4.15 $$\begin{aligned} \alpha _n^0\ge (1-\varepsilon )\alpha _0\quad \text {and} \quad \alpha _n^1\ge (1-\varepsilon )\alpha _1. \end{aligned}$$

In particular, in view of (4.6) we have

4.16 $$\begin{aligned} (1-\varepsilon )\min \{\alpha _0,\alpha _1\}\sup \left\{ \left\| u_n\right\| _{BPGV^2_{{\mathscr {B}}_n}(Q)}: \,\, n\in {\mathbb {N}}\right\} <+\infty . \end{aligned}$$

Since ${\mathscr {B}}_n\rightarrow {\mathscr {B}}$ in $\ell ^{\infty }$, choosing $r>0$ small enough there exists $N>0$ such that ${\mathscr {B}}_n\subset ({\mathscr {B}})_1$ for all $n\ge N$. Thus, by (4.8) and (4.16), we infer that

$$\begin{aligned} \sup \left\{ \left\| u_n\right\| _{BV(Q)}:\,\, n\in {\mathbb {N}}\right\} \le C_1 \sup \left\{ \left\| u_n\right\| _{BPGV^2_{{\mathscr {B}}_n}(Q)}:\,\, n\in {\mathbb {N}}\right\} <+\infty , \end{aligned}$$

and thus we may find $u\in BV(Q)$ such that, up to a subsequence (not relabeled), $u_n\mathrel {\mathop {\rightharpoonup }\limits ^{*}}u$ in *BV*(*Q*).

Additionally, again from Proposition 3.8, for every $n\in {\mathbb {N}}$ there exists $v_n\in BV_{{\mathscr {B}}_n}(Q;{{{\mathbb {R}}}^N})$ such that,

$$\begin{aligned} PGV_{\alpha _n,{\mathscr {B}}_n}^2(u_n)=\alpha _n^0\left|Du_n-v_{n}\right|_{{\mathcal {M}_b}(Q;{\mathbb {R}}^N)} +\alpha _n^1\left|{\mathscr {B}}_n v_n\right|_{{\mathcal {M}_b}(Q;{{\mathbb {R}}^{N\times N}})}. \end{aligned}$$

By (4.6) and (4.7), and in view of Assumption 3.3, we find $v\in BV_{ {\mathscr {B}}}(Q;{{{\mathbb {R}}}^N})$ such that, up to a subsequence (not relabeled), $v_n\rightarrow v$ strongly in $L^1$. Therefore, we have

$$\begin{aligned} {\liminf _{n\rightarrow \infty }}PGV_{\alpha _n,{\mathscr {B}}_n}^2(u_n)&\ge {\liminf _{n\rightarrow \infty }}\alpha _n^0\left|Du_n-v_{n}\right|_{{\mathcal {M}_b}(Q;{\mathbb {R}}^N)}+{\liminf _{n\rightarrow \infty }}\alpha _n^1\left|{\mathscr {B}}_n v_n\right|_{{\mathcal {M}_b}(Q;{{\mathbb {R}}^{N\times N}})}\\&\ge (1-\varepsilon )\alpha _0\left|D u- v\right|_{{\mathcal {M}_b}(Q;{\mathbb {R}}^N)} +(1-\varepsilon )\alpha _1\left|{\mathscr {B}}v\right|_{{\mathcal {M}_b}(Q;{{\mathbb {R}}^{N\times N}})}\\&\ge (1-\varepsilon )PGV^2_{\alpha ,{\mathscr {B}}}( u), \end{aligned}$$

where in the second to last inequality we used Assumption 3.3 and (4.15). The arbitrariness of $\varepsilon$ concludes the proof of the proposition. $\square$

### Proposition 4.5

Let $\left\{ {{\mathscr {B}}_n}\right\} _{n=1}^\infty \subset \Pi$ satisfy Assumption 3.3, and let $\left\{ {\alpha _n}\right\} _{n=1}^\infty \subset {\mathbb {R}}^{2}_+$ be such that ${\mathscr {B}}_n\rightarrow {\mathscr {B}}$ in $\ell ^{\infty }$ and $\alpha _n\rightarrow \alpha \in {\mathbb {R}}^{2}_+$. Then for every $u\in BV(Q)$ there exists $\left\{ {u_n}\right\} _{n=1}^\infty \subset BV(Q)$ such that $u_n\rightarrow u$ in $L^1(Q)$ and

$$\begin{aligned} \limsup _{n\rightarrow \infty }{PGV_{\alpha _n,{\mathscr {B}}_n}^{2}(u_n)} \le {PGV_{\alpha ,{\mathscr {B}}}^{2}(u)}. \end{aligned}$$

### Proof

This is a direct consequence of Proposition 3.9 by choosing $u_n:=u$. $\square$

We close Sect. 4 by proving Theorem 4.2.

### Proof of Theorem 4.2

Properties (Compactness) and (Liminf inequality) hold in view of Proposition 4.4, and Property (Recovery sequence) follows from Proposition 4.5. $\square$

## The Bilevel Training Scheme with *PGV*-Regularizers

In this section, we introduce a bilevel training scheme associated to our class of regularizers and show its well-posedness. Let $u_\eta \in L^2(Q)$ and $u_c\in BV(Q)$ be the corrupted and clean images, respectively. In what follows we will refer to pairs $(u_c,u_\eta )$ as training pairs. We recall that $\Pi$ was introduced in Definition 3.4.

### Definition 5.1

We say that $\Sigma \subset \Pi$ is a training set if the operators in $\Sigma$ satisfy Assumption 3.3, and if $\Sigma$ is closed and bounded in $\ell ^{\infty }$.

Examples of training sets are provided in Sect. 7. We introduce the following bilevel training scheme.

### Definition 5.2

Let $\theta \in (0,1)$ and let $\Sigma$ be a training set. The two levels of the scheme $({\mathcal {T}}^{2}_\theta )$are 
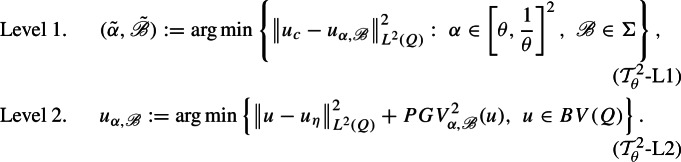


We first show that the Level 2 problem in (${\mathcal {T}}^2_\theta$-L2) admits a solution for every given $u_\eta \in L^2(Q)$, and for every $\alpha \in {\mathbb {R}}^2_+$.

### Proposition 5.3

Let $u_\eta \in L^2(Q)$. Let ${\mathscr {B}}\in \Sigma$, and let $\alpha \in {\mathbb {R}}^2_+$. Then there exists $u_{\alpha ,{\mathscr {B}}}\in BV(Q)$ such that

$$\begin{aligned} \Vert {u_{\alpha ,{\mathscr {B}}}}-u_\eta \Vert ^2_{L^2(Q)}+PGV^2_{\alpha ,{\mathscr {B}}}({u_{\alpha ,{\mathscr {B}}}}) =\min \left\{ \left\| u-u_\eta \right\| ^2_{L^2(Q)}+PGV^2_{\alpha ,{\mathscr {B}}}(u): \, u\in BV(Q)\right\} . \end{aligned}$$

### Proof

Without loss of generality, we assume that $\alpha :=(1,1)$. Let $\left\{ {u_n}\right\} _{n=1}^\infty \subset BV(Q)$ be such that

5.1 $$\begin{aligned} \left\| u_n-u_{\eta }\right\| ^2_{L^2(Q)}+{PGV_{{\mathscr {B}}}^{2}}(u_n) \le \inf \left\{ \Vert u-u_{\eta }\Vert ^2_{L^2(Q)}+{PGV_{{\mathscr {B}}}^{2}}(u):\, u\in BV(Q)\right\} +1/n, \end{aligned}$$

for every $n\in {\mathbb {N}}$, and let $\{v_n\}\subset BV_{{\mathscr {B}}}(Q)$ be the associated sequence of maps provided by Proposition 3.8. In view of (5.1), there exists a constant *C* such that

5.2 $$\begin{aligned} \Vert u_n-u_\eta \Vert ^2_{L^2(Q)}+\left|Du_n-v_n\right|_{{\mathcal {M}_b}(Q;{{{\mathbb {R}}}^N})} +\left|{\mathscr {B}}v_n\right|_{{\mathcal {M}_b}(Q;{{\mathbb {R}}^{N\times N}})}\le C \end{aligned}$$

for every $n\in {\mathbb {N}}$. We claim that

5.3 $$\begin{aligned} \sup \left\{ \Vert v_n\Vert _{L^1(Q;{\mathbb {R}}^N)}:\,\, n\in {\mathbb {N}}\right\} <+\infty . \end{aligned}$$

Indeed, if (5.3) does not hold, then, up to the extraction of a subsequence (not relabeled), we have

$$\begin{aligned} \lim _{n\rightarrow +\infty }\left\| v_n\right\| _{L^1(Q;{{{\mathbb {R}}}^N})}= +\infty . \end{aligned}$$

Setting

5.4 $$\begin{aligned} \tilde{u}_n:=\frac{u_n}{\Vert v_n\Vert _{L^1(Q;{{{\mathbb {R}}}^N})}}\quad \text {and } \quad \tilde{v}_n:=\frac{v_n}{\Vert v_n\Vert _{L^1(Q;{{{\mathbb {R}}}^N})}} \quad \text {for every }n\in {\mathbb {N}}, \end{aligned}$$

and dividing both sides of (5.2) by $\Vert v_n\Vert _{L^1(Q)}$, we deduce that

5.5 $$\begin{aligned} \lim _{n\rightarrow +\infty }\left[ {\left\| \tilde{u}_n -\frac{u_{\eta }}{\Vert v_n\Vert _{L^1(Q;{{{\mathbb {R}}}^N})}}\right\| ^2_{L^2(Q)} +\left|D\tilde{u}_n-\tilde{v}_n\right|_{{\mathcal {M}_b}(Q;{{{\mathbb {R}}}^N})} +\left|{\mathscr {B}}\tilde{v}_n\right|_{{\mathcal {M}_b}(Q;{{\mathbb {R}}^{N\times N}})}}\right] =0. \end{aligned}$$

In view of (5.4) and (5.5), and by Assumption 3.3, there exists $\tilde{v}\in BV_{{\mathscr {B}}}(Q;{{{\mathbb {R}}}^N})$, with

5.6 $$\begin{aligned} \Vert \tilde{v}\Vert _{L^1(Q;{\mathbb {R}}^N)}=1, \end{aligned}$$

such that

5.7 $$\begin{aligned} \tilde{v}_n\rightarrow \tilde{v}\quad \text {strongly in }L^1(Q;{\mathbb {R}}^N), \end{aligned}$$

and

$$\begin{aligned} {\mathscr {B}}\tilde{v}_n\mathrel {\mathop {\rightharpoonup }\limits ^{*}}{\mathscr {B}}\tilde{v}\quad \text {weakly}^*\text { in }{\mathcal {M}_b}(Q;{{\mathbb {R}}^{N\times N}}). \end{aligned}$$

Additionally, (5.5) and (5.7) yield

5.8 $$\begin{aligned} \tilde{u}_n\rightarrow 0\quad \text {strongly in }L^2(Q), \end{aligned}$$

and

5.9 $$\begin{aligned} \limsup _{n\rightarrow +\infty } \left|D\tilde{u}_n-\tilde{v}\right|_{{\mathcal {M}_b}(Q;{{{\mathbb {R}}}^N})} \le \lim _{n\rightarrow +\infty }\left|D\tilde{u}_n-\tilde{v}_n\right|_{{\mathcal {M}_b}(Q;{{{\mathbb {R}}}^N})} +\lim _{n\rightarrow +\infty }\left\| \tilde{v}_n-\tilde{v}\right\| _{L^1(Q;{{{\mathbb {R}}}^N})}=0. \end{aligned}$$

Since by (5.8) $D\tilde{u}_n\rightarrow 0$ in the sense of distribution, we deduce from (5.9) that $\tilde{v} =0$. This contradicts (5.6), and implies claim (5.3).

By combining (5.2) and (5.3), we obtain the uniform bound

$$\begin{aligned} \left|Du_n\right|_{{\mathcal {M}_b}(Q;{{{\mathbb {R}}}^N})}\le \left|Du_n-v_n\right|_{{\mathcal {M}_b}(Q;{{{\mathbb {R}}}^N})} +\Vert v_n\Vert _{L^1(Q;{\mathbb {R}}^N)}\le C \end{aligned}$$

for every $n\in {\mathbb {N}}$ and some $C>0$. Thus, by (5.2) and Assumption 3.2 there exist $u_{\mathscr {B}}\in BV(Q)$ and $v\in BV_{{\mathscr {B}}}(Q)$ such that, up to the extraction of a subsequence (not relabeled),

$$\begin{aligned}&u_n\rightharpoonup u_{\mathscr {B}}\quad \text {weakly in }L^2(Q),\\&u_n\mathrel {\mathop {\rightharpoonup }\limits ^{*}}u_{\mathscr {B}}\quad \text {weakly}^*\text { in }BV(Q),\\&v_n\rightarrow v\quad \text {strongly in }L^1(Q;{\mathbb {R}}^N),\\&{\mathscr {B}}v_n\mathrel {\mathop {\rightharpoonup }\limits ^{*}}{\mathscr {B}}v\text { weakly}^*\text { in }{\mathcal {M}_b}(Q;{{\mathbb {R}}^{N\times N}}). \end{aligned}$$

In view of (5.1), and by lower-semicontinuity, we obtain the inequality

$$\begin{aligned}&\left\| u_{\mathscr {B}}-u_0\right\| ^2_{L^2(Q)}+\left|Du_{\mathscr {B}}-v\right|_{{\mathcal {M}_b}(Q;{{{\mathbb {R}}}^N})} +\left|{\mathscr {B}}v\right|_{{\mathcal {M}_b}(Q;{{\mathbb {R}}^{N\times N}})}\\&\quad \le \inf \left\{ \left\| u-u_\eta \right\| ^2_{L^2(Q)} +PGV_{\mathscr {B}}^2(u):\, u\in BV(Q)\right\} . \end{aligned}$$

$$\square $$

### Theorem 5.4

The training scheme $({\mathcal {T}}^{2}_\theta )$ admits at least one solution $({\tilde{\alpha }}, \tilde{{\mathscr {B}}})\in \big [\theta ,1/\theta ]^{2}\times \Sigma$, and provides an associated optimally reconstructed image $u_{{\tilde{\alpha }},\tilde{{\mathscr {B}}}}\in BV(Q)$.

### Proof

By the boundedness and closedness of $\Sigma$ in $\ell ^{\infty }$, up to a subsequence (not relabeled), there exists $({\tilde{\alpha }}, \tilde{{\mathscr {B}}})\in [\theta ,1/\theta ]^2\times \Sigma$ such that $\alpha _n\rightarrow {\tilde{\alpha }}$ in ${\mathbb {R}}^2$ and ${\mathscr {B}}_n\rightarrow \tilde{{\mathscr {B}}}$ in $\ell ^{\infty }$. Therefore, in view of Theorem 4.2 and the Fundamental Theorem of $\Gamma$-convergence (see, e.g. Dal Maso 1993), we have

5.10 $$\begin{aligned} u_{\alpha _n,{\mathscr {B}}_n}\mathrel {\mathop {\rightharpoonup }\limits ^{*}}u_{{\tilde{\alpha }},\tilde{{\mathscr {B}}}} \text { weakly}^*\text { in }BV(Q) \text { and strongly in }L^1(Q), \end{aligned}$$

where $u_{\alpha _n,{\mathscr {B}}_n}$ and $u_{{\tilde{\alpha }},\tilde{{\mathscr {B}}}}$ are defined in (${\mathcal {T}}^2_\theta$-L2).

By (5.10), we have

$$\begin{aligned} \left\| u_{{\tilde{\alpha }},\tilde{{\mathscr {B}}}}-u_c\right\| _{L^2(Q)} \le {\liminf _{n\rightarrow \infty }}\left\| u_{\alpha _n,{\mathscr {B}}_n}-u_c\right\| _{L^2(Q)}, \end{aligned}$$

which completes the proof. $\square$

## Training Set $\Sigma [\mathscr {A}]$ Based on $(\mathscr {A},{\mathscr {B}})$ Training Operators Pairs

This section is devoted to providing a class of operators ${\mathscr {B}}$ belonging to $\Pi$ (see Definition 3.4), satisfying Assumption 3.3, and being closed with respect to the convergence in (2.2). Recall that $Q=\left( -\tfrac{1}{2},\tfrac{1}{2}\right) ^N$.

### A Subcollection of $\Pi$ Characterized by $(\mathscr {A},{\mathscr {B}})$ Training Operators Pairs

Let *U* be an open set in ${\mathbb {R}}^{N}$, and let $\mathscr {A}:{\mathcal {D}}'(U;{{{\mathbb {R}}}^N})\rightarrow {\mathcal {D}}'(U;{{{\mathbb {R}}}^N})$ be a *d*-th order differential operator, defined as

$$\begin{aligned} \mathscr {A}u:= \sum _{\left|a\right|\le d}A_{a} \frac{\partial ^a}{\partial x^a}u \quad \text {for every }\,u\in {\mathcal {D}}'(U;{{{\mathbb {R}}}^N}), \end{aligned}$$

where, for every multi-index $a=(a^1,a^{2},\ldots ,a^N)\in {\mathbb {N}}^N$,

$$\begin{aligned} \frac{\partial ^a}{\partial x^a}:=\frac{\partial ^{a^1}}{\partial x_1^{a^1}}\frac{\partial ^{a^{2}}}{\partial x_2^{a^{2}}}\cdots \frac{\partial ^{a^N}}{\partial x_N^{a^N}} \end{aligned}$$

is meant in the sense of distributional derivatives, and $A_{a}$ is a linear operator mapping from ${{{\mathbb {R}}}^N}$ to ${{{\mathbb {R}}}^N}$. Let ${\mathscr {B}}$ be a first order differential operator, ${\mathscr {B}}:{\mathcal {D}}'(U;{\mathbb {R}}^N)\rightarrow {\mathcal {D}}'(U;{\mathbb {R}}^{N\times N})$, given by

$$\begin{aligned} \mathscr {B} v:=\sum _{i=1}^N B^i\frac{\partial }{\partial x_i} v \quad \text {for every }v\in {\mathcal {D}}'(U;{\mathbb {R}}^N), \end{aligned}$$

where $B^i\in {\mathbb {M}}^{N^{3}}$ for each $i=1,\dots ,N$, and where $\frac{\partial }{\partial x_i}$ denotes the distributional derivative with respect to the *i*-th variable. We will restrict our analysis to elliptic pairs $(\mathscr {A},{\mathscr {B}})$ satisfying the ellipticity assumptions below.

#### Definition 6.1

We say that $(\mathscr {A},{\mathscr {B}})$ is a training operator pair if ${\mathscr {B}}$ has finite dimensional null-space ${{\mathcal {N}}}({\mathscr {B}})$, and $(\mathscr {A},{\mathscr {B}})$ satisfies the following assumptions:

1. For every $\lambda \in \left\{ -1,1\right\} ^N$, the operator $\mathscr {A}$ has a fundamental solution $P_{\lambda }\in L^1({{{\mathbb {R}}}^N};{{{\mathbb {R}}}^N})$ such that:
  - $\mathscr {A}P_{\lambda } = \lambda \delta$, where $\delta$ denotes the Dirac measure centered at the origin;
  - $P_{\lambda }\in C^{\infty }({{{\mathbb {R}}}^N}\setminus \{0\};{{{\mathbb {R}}}^N})$ and $\frac{\partial ^{a}}{\partial x^{a}} P_{\lambda }\in L^1_\textrm{loc}({{{\mathbb {R}}}^N};{{{\mathbb {R}}}^N})$ for every multi-index $a\in {\mathbb {N}}^N$ with $|a|\le d-1$ (where *d* is the order of the operator ${\mathscr {A}}$).
2. For every open set $U\subset {\mathbb {R}}^N$ such that $Q\subset U$, and for every $u\in W^{{d-1},1}(U;{{{\mathbb {R}}}^N})$ and $v\in C^{\infty }_c(U;{{{\mathbb {R}}}^N})$
  6.1 $$\begin{aligned} \left\| ({\mathscr {A}}u)_i*v_i\right\| _{L^1(U)}\le C_{\mathscr {A}}\left[ \sum _{\left|a\right|\le d-1} \left\| \frac{\partial ^{a}}{\partial x^{a}}u\right\| _{L^1(U;{{{\mathbb {R}}}^N})}\right] \left|{\mathscr {B}}v\right|_{{\mathcal {M}}_b(U;{{\mathbb {R}}^{N\times N}})}, \end{aligned}$$
  for every $i=1,\ldots , N$, where the constant $C_{\mathscr {A}}$ depends only on the operator $\mathscr {A}$. The same property holds for $u\in C^{\infty }_c(U;{{{\mathbb {R}}}^N})$ and $v\in BV_{{\mathscr {B}}}(U;{{{\mathbb {R}}}^N})$ (see (3.1)).

Note that, in view of 1*b*. one has, directly, the following property: for every $a\in {\mathbb {N}}^N$ with $|a|\le d-1$, and for every open set $U\subset {\mathbb {R}}^N$ such that $Q\subset U$, we have

6.2 $$\begin{aligned} \sum _{\left|a\right|= d-1} \left\| \tau _h\left( \frac{\partial ^{a}}{\partial x^{a}} {P_{\lambda }}\right) -\frac{\partial ^{a}}{\partial x^{a}} P_{\lambda }\right\| _{L^1(U;{\mathbb {R}}^N)} =:M_\mathscr {A}(U;h)\rightarrow 0\quad \text {as}\, \,\left|h\right|\rightarrow 0, \end{aligned}$$

where for $h\in {{{\mathbb {R}}}^N}$, the translation operator $\tau _h:L^1({{{\mathbb {R}}}^N};{{{\mathbb {R}}}^N})\rightarrow L^1({{{\mathbb {R}}}^N};{{{\mathbb {R}}}^N})$ is defined by

6.3 $$\begin{aligned} \tau _h w(x):=w(x+h)\quad \text {for every }w \in L^1({{{\mathbb {R}}}^N};{{{\mathbb {R}}}^N})\,\text {and for a.e.}\, x\in {{{\mathbb {R}}}^N}. \end{aligned}$$

Explicit examples of operators $\mathscr {A}$ and ${\mathscr {B}}$ satisfying Definition 6.1 are provided in Sect. 7. Condition 2. in Definition 6.1 can be interpreted as an “integration by parts-requirement”, as highlighted by the example below. Let $N=2$, $d=2$, ${\mathscr {B}}=\nabla$, and let $U\subset {\mathbb {R}}^2$ be an open set such that $Q\subset U$. Consider the following second order differential operator

$$\begin{aligned} {\mathscr {A}}u:=\left( \frac{\partial ^2 u_1}{\partial x_1^2} \quad \frac{\partial ^2 u_2}{\partial x_2^2}\right) ^{\intercal } \quad \text {for every}\quad u=(u_1,u_2)^{\intercal }\in D'(U;{\mathbb {R}}^2). \end{aligned}$$

Then, for every $u\in W^{2,1}(U;{\mathbb {R}}^2)$ and $v\in C^{\infty }_c(U;{\mathbb {R}}^2)$ there holds

$$\begin{aligned} \left\| ({\mathscr {A}}u)_i*v_i\right\| _{L^1(U)}&=\left\| \frac{\partial ^2 u_i}{\partial x_i^2} *v_i\right\| _{L^1(U)}=\left\| \frac{\partial u_i}{\partial x_i} *\frac{\partial v_i}{\partial x_i}\right\| _{L^1(U)}\\&\le \left\| \nabla u\right\| _{L^1(U;{\mathbb {R}}^{2\times 2})} \left\| \nabla v\right\| _{L^1(U;{\mathbb {R}}^{2\times 2})}\\&=\left\| \nabla u\right\| _{L^1(U;{\mathbb {R}}^{2\times 2})} \left\| {\mathscr {B}}v\right\| _{L^1(U;{\mathbb {R}}^{2\times 2})}, \end{aligned}$$

for every $i=1,2$. In other words, the pair $({\mathscr {A}},{\mathscr {B}})$ satisfies (6.1) with $C_{{\mathscr {A}}}=1$.

#### Definition 6.2

For every $\mathscr {A}$ as in Definition 6.1 we denote by $\Pi _\mathscr {A}$ the following collection of first order differential operators ${\mathscr {B}}$,

$$\begin{aligned} \Pi _\mathscr {A}:=\left\{ {\mathscr {B}}:\,\, (\mathscr {A},{\mathscr {B}})\ \text {is a training operator pair}\right\} . \end{aligned}$$

The following extension result in $BV_{{\mathscr {B}}}$ is a corollary of the properties of the trace operator defined in Breit et al. (2017, Section 4).

#### Lemma 6.3

Let ${\mathscr {B}}\in \Pi _\mathscr {A}$, and let $BV_{\mathscr {B}}(Q;{{{\mathbb {R}}}^N})$ be the space introduced in Definition 3.1. Then there exists a continuous extension operator ${{\mathbb {T}}}:\,BV_{\mathscr {B}}(Q;{{{\mathbb {R}}}^N})\rightarrow BV_{\mathscr {B}}({{{\mathbb {R}}}^N};{{{\mathbb {R}}}^N})$ such that ${{\mathbb {T}}}u=u$ almost everywhere in *Q* for every $u\in BV_{\mathscr {B}}(Q;{{{\mathbb {R}}}^N})$.

#### Proof

Since ${{\mathcal {N}}}({\mathscr {B}})$ is finite dimensional, in view of Breit et al. (2017, (4.9) and Theorem 1.1) there exists a continuous trace operator $\textrm{tr}\,:\,BV_{\mathscr {B}}(Q;{{{\mathbb {R}}}^N})\rightarrow L^1(\partial Q;{{{\mathbb {R}}}^N})$. By the classical results by E. Gagliardo (see Gagliardo (1957)) there exists a linear and continuous extension operator $\textrm{E}: L^1(\partial Q;{{{\mathbb {R}}}^N})\rightarrow W^{1,1}({\mathbb {R}}^N{\setminus } Q;{{{\mathbb {R}}}^N})$. The statement follows by setting

6.4 $$\begin{aligned} {{\mathbb {T}}}u:=u\chi _{Q}+\textrm{E}(\textrm{tr}(u))\chi _{{\mathbb {R}}^N\setminus Q}, \end{aligned}$$

where $\chi _{Q}$ and $\chi _{{\mathbb {R}}^N{\setminus } Q}$ denote the characteristic functions of the sets *Q* and ${\mathbb {R}}^N\setminus Q$, respectively, and by Theorem Breit et al. (2017, Corollary 4.21). $\square$

#### Remark 6.4

We point out that, as a direct consequence of Lemma 6.3, we obtain

6.5 $$\begin{aligned} \left|{\mathscr {B}}({{\mathbb {T}}}u)\right|_{{\mathcal {M}_b}({{{\mathbb {R}}}^N};{{\mathbb {R}}^{N\times N}})} \le C_{{\mathscr {B}}} {\left\| u\right\| _{BV_{{\mathscr {B}}}(Q;{{{\mathbb {R}}}^N})}}. \end{aligned}$$

In particular, from (6.4) and Theorem Breit et al. (2017, Corollary 4.21), the constant $C_{\mathscr {B}}$ in the inequality above is obtained by the following estimate:

$$\begin{aligned}&\left|{\mathscr {B}}({{\mathbb {T}}}(u))\right|_{{\mathcal {M}_b}({{{\mathbb {R}}}^N};{{\mathbb {R}}^{N\times N}})} \le \left|{\mathscr {B}}u\right|_{{\mathcal {M}_b}(Q;{{\mathbb {R}}^{N\times N}})}+\left|{\mathscr {B}}(\textrm{E} (\textrm{tr}(u)))\right|_{{\mathcal {M}_b}({{{\mathbb {R}}}^N}\setminus Q;{{\mathbb {R}}^{N\times N}})}\\&\quad \le \left|{\mathscr {B}}u\right|_{{\mathcal {M}_b}(Q;{{\mathbb {R}}^{N\times N}})}+\left\| {\mathscr {B}}\right\| _{\ell ^\infty } C_{\textrm{G}}\Vert \textrm{tr}(u)\Vert _{L^1(\partial Q;{{{\mathbb {R}}}^N})} \le (1+\left\| B\right\| _{\ell ^\infty }C_G C_{T_{\mathscr {B}}})\Vert u\Vert _{BV_{\mathscr {B}}(Q)}, \end{aligned}$$

where $C_{\textrm{G}}$ is the constant associated to the classical Gagliardo’s extension in $W^{1,1}$ (see Gagliardo 1957) and is thus independent of ${\mathscr {B}}$, whereas $C_{T_{\mathscr {B}}}$ is the constant associated to the trace operator in $BV_{\mathscr {B}}$. Hence, $C_{{\mathscr {B}}}=(1+\left\| B\right\| _{\ell ^\infty }C_G C_{T{\mathscr {B}}}))$.

The main result of this section is the following.

#### Theorem 6.5

Let $\mathscr {A}$ be as in Definition 6.1. Let $\Pi$ and $\Pi _\mathscr {A}$ be the collections of first order operators introduced in Definitions 3.4 and 6.2, respectively. Then every operator ${\mathscr {B}}\in \Pi _\mathscr {A}$ satisfies Assumption 3.2. Additionally, every subset of operators in $\Pi _{\mathscr {A}}$ for which the constants in (6.5) are uniformly bounded fulfills Assumption 3.3.

We proceed by first recalling two preliminary results from the literature. The next proposition, that may be found in Brezis (2011, Theorem 4.26), will be instrumental in the proof of a regularity result for distributions with bounded ${\mathscr {B}}$-total-variation (see Proposition 6.9).

#### Proposition 6.6

Let ${\mathcal {F}}$ be a bounded set in $L^p({{{\mathbb {R}}}^N})$ with $1\le p<+\infty$. Assume that

$$\begin{aligned} \lim _{\left|h\right|\rightarrow 0}\left\| \tau _h f-f\right\| _{L^p({{{\mathbb {R}}}^N})}=0 \text { uniformly in }{\mathcal {F}}. \end{aligned}$$

Then, denoting by ${\mathcal {F}}\lfloor _{Q}$ the collection of the restrictions to *Q* of the functions in ${\mathcal {F}}$, the closure of ${\mathcal {F}}\lfloor _{Q}$ in $L^p(Q)$ is compact.

We also recall some basic properties of the space $BV_{\mathscr {B}}(Q;{{{\mathbb {R}}}^N})$ for ${\mathscr {B}}\in \Pi _\mathscr {A}$ [see Definition 3.1 and Breit et al. (2017, Section 2)].

#### Proposition 6.7

Let ${\mathscr {B}}\in \Pi _{\mathscr {A}}$. Let *U* be an open set in ${\mathbb {R}}^N$. Then

1. $BV_{\mathscr {B}}(U;{{{\mathbb {R}}}^N})$ is a Banach space with respect to the norm defined in (3.2);
2. $C^\infty (U,{{{\mathbb {R}}}^N})$ is dense in $BV_{\mathscr {B}}(U;{{{\mathbb {R}}}^N})$ in the strict topology, i.e., for every $u\in BV_{\mathscr {B}}(U;{{{\mathbb {R}}}^N})$ there exists $\left\{ {u_n}\right\} _{n=1}^\infty \subset C^\infty (U,{{{\mathbb {R}}}^N})$ such that
  $$\begin{aligned} u_n\rightarrow u\text { strongly in }L^1(U;{{{\mathbb {R}}}^N})\text { and } \left|{\mathscr {B}}u_n\right|_{{\mathcal {M}}_b(U;{{\mathbb {R}}^{N\times N}})}\rightarrow \left|{\mathscr {B}}u\right|_{{\mathcal {M}}_b(U;{{\mathbb {R}}^{N\times N}})}. \end{aligned}$$

Before we establish Theorem 6.5, we prove a technical lemma.

#### Lemma 6.8

Let $k\in {\mathbb {N}}$. Then there exists a constant $C>0$ such that, for every $h\in {{{\mathbb {R}}}^N}$ and $w\in W_{\textrm{loc}}^{k,1}({{{\mathbb {R}}}^N};{{{\mathbb {R}}}^N})$, there holds

$$\begin{aligned}&\limsup _{\left|h\right|\rightarrow 0}\sum _{\left|a\right|\le k}\left\| \tau _h \Big (\frac{\partial ^{a}}{\partial x^{a}}w\Big ) -\frac{\partial ^{a}}{\partial x^{a}}w\right\| _{L^1(Q;{{{\mathbb {R}}}^N})}\\&\quad \le \limsup _{\left|h\right|\rightarrow 0}C \sum _{\left|a\right|= k} \left\| \tau _h\Big (\frac{\partial ^{a}}{\partial x^{a}}w\Big ) -\frac{\partial ^{a}}{\partial x^{a}}w\right\| _{L^1(Q;{{{\mathbb {R}}}^N})}, \end{aligned}$$

where $\tau _h$ is the operator defined in (6.3).

#### Proof

By the linearity of $\tau _h$, we have

$$\begin{aligned} \tau _h\Big (\frac{\partial ^{a}}{\partial x^{a}}w\Big ) -\frac{\partial ^{a}}{\partial x^{a}}w =\frac{\partial ^{a}}{\partial x^{a}}(\tau _hw-w). \end{aligned}$$

On the one hand, by the Sobolev embedding theorem (see, e.g., Leoni 2009), we have

6.6 $$\begin{aligned}&\sum _{\left|a\right|\le k}\left\| \tau _h\Big (\frac{\partial ^{a}}{\partial x^{a}}w\Big )-\frac{\partial ^{a}}{\partial x^{a}}w\right\| _{L^1(Q;{{{\mathbb {R}}}^N})}\nonumber \\&\quad =\sum _{\left|a\right|\le k}\left\| \frac{\partial ^{a}}{\partial x^{a}} (\tau _hw-w)\right\| _{L^1(Q;{{{\mathbb {R}}}^N})}\nonumber \\&\quad \le C\left\| \tau _h(w)-w\right\| _{L^1(Q;{{{\mathbb {R}}}^N})}+C\sum _{\left|a\right|= k} \left\| \frac{\partial ^{a}}{\partial x^{a}}(\tau _hw-w)\right\| _{L^1(Q;{{{\mathbb {R}}}^N})}. \end{aligned}$$

On the other hand, by the continuity of the translation operator in $L^1$ (see, e.g., Brezis (2011, Lemma 4.3) for a proof in ${\mathbb {R}}^N$, the analogous argument holds on bounded open sets) we have

6.7 $$\begin{aligned} \limsup _{\left|h\right|\rightarrow 0} \left\| \tau _h(w)-w\right\| _{L^1(Q;{{{\mathbb {R}}}^N})}=0. \end{aligned}$$

The result follows by combining (6.6) and (6.7). $\square$

The next proposition shows that operators in $\Pi _\mathscr {A}$ satisfy Assumption 3.2.

#### Proposition 6.9

Let ${\mathscr {B}}\in \Pi _\mathscr {A}$, and let $BV_{\mathscr {B}}(Q;{{{\mathbb {R}}}^N})$ be the space introduced in Definition 3.1. Then the injection of $BV_{\mathscr {B}}(Q;{{{\mathbb {R}}}^N})$ into $L^1(Q;{{{\mathbb {R}}}^N})$ is compact.

#### Proof

For every $u\in BV_{\mathscr {B}}(Q;{\mathbb {R}}^N)$ we still denote by *u* its extension to $BV_{\mathscr {B}}(2Q;{\mathbb {R}}^N)$ provided by Lemma 6.3. In view of Proposition 6.7, for every $u\in BV_{\mathscr {B}}(Q;{\mathbb {R}}^N)$ we then find a sequence of maps $\{ v^n_u \}_{n=1}^{\infty }\subset C^{\infty }(2Q;{\mathbb {R}}^N)$ such that

6.8 $$\begin{aligned} \Vert v^n_u -u\Vert _{L^1({2}Q;{\mathbb {R}}^N)}+\left|\left\| {\mathscr {B}}v^n_u \right\| _{L^1({2}Q;{{\mathbb {R}}^{N\times N}})} -\left|{\mathscr {B}}u\right|_{{\mathcal {M}}_b({2}Q;{{\mathbb {R}}^{N\times N}})}\right|\le \frac{1}{n}. \end{aligned}$$

With a slight abuse of notation, we still denote by $$v^n_u $$ the $$C^d$$-extension of the above maps to the whole $${\mathbb {R}}^N$$ (see e.g. Fefferman 2007), where *d* is the order of the operator $$\mathscr {A}$$. Without loss of generality, up to a multiplication by a cut-off function, we can assume that $$v^n_u \in C^d_c({3}Q;{\mathbb {R}}^N)$$ for every $$n\in {\mathbb {N}}$$.

We first show that, setting

$$\begin{aligned} {\mathcal {F}}:=\left\{ u\in L^1(Q;{{{\mathbb {R}}}^N}): \,\, \left\| u\right\| _{BV_{\mathscr {B}}(Q;{{{\mathbb {R}}}^N})}\le 1\right\} , \end{aligned}$$

for every $n\in {{\mathbb {N}}}$ there holds

6.9 $$\begin{aligned} \lim _{\left|h\right|\rightarrow 0}\sup _{u\in {\mathcal {F}}} \left\{ \left\| \tau _h {v}^n_u-{v}^n_u\right\| _{L^1({2}Q;{{{\mathbb {R}}}^N})}\right\} =0, \end{aligned}$$

where we recall $$\tau _h$$ from Theorem 6.6, and where for fixed $$u\in {\mathcal {F}}$$, $$v^n_u $$ is as above and satisfying (6.8).

Let $h\in {{{\mathbb {R}}}^N}$ and let $\delta _h$ be the Dirac distribution centered at $h\in {{{\mathbb {R}}}^N}$. By the properties of the fundamental solution $P_{\lambda }$ we deduce

$$\begin{aligned} \tau _h(\lambda _i v^n_{u,i} )&= \delta _h*\lambda _i v^n_{u,i} =\delta _h*(\lambda _i\delta *v^n_{u,i} )=\delta _h*\left( (\mathscr {A}{P_{\lambda }})_i*v^n_{u,i} \right) \\&=\left( \delta _h*(\mathscr {A}{P_{\lambda }})_i\right) *v^n_{u,i} =\left( \mathscr {A}\left( \delta _h*({P_{\lambda }})\right) \right) _i*v^n_{u,i} , \end{aligned}$$

for every $i=1,\ldots , N$, and every $\lambda \in \left\{ -1,1\right\} ^N$. Therefore, we obtain that

6.10 $$\begin{aligned}&\left\| \tau _h( \lambda _i v^n_{u,i}) -\lambda _i v^n_{u,i} \right\| _{L^1({2}Q;{{{\mathbb {R}}}^N})}\nonumber \\&=\left\| \left( \mathscr {A}\left( \delta _h*({P_{\lambda }})\right) \right) _i *v^n_{u,i} -\left( \mathscr {A}{P_{\lambda }}\right) _i*v^n_{u,i} \right\| _{L^1({2}Q;{{{\mathbb {R}}}^N})}\nonumber \\&=\left\| \left( \mathscr {A}\left( \delta _h*({P_{\lambda }})-{P_{\lambda }}\right) \right) _i *v^n_{u,i} \right\| _{L^1({2}Q;{{{\mathbb {R}}}^N})}\nonumber \\&\le C_\mathscr {A}\left[ \sum _{\left|a\right|\le d-1} \left\| \tau _h \left( \frac{\partial ^{a}}{\partial x^{a}}{P_{\lambda }}\right) -\frac{\partial ^{a}}{\partial x^{a}}{P_{\lambda }}\right\| _{L^1({2}Q;{{{\mathbb {R}}}^N})}\right] \left|{\mathscr {B}}v^n_u \right|_{{\mathcal {M}}_b({2}Q;{{\mathbb {R}}^{N\times N}})} \end{aligned}$$

for every $\lambda \in \{-1,1\}^N$, where in the last inequality we used the fact that $\tau _h{P_{\lambda }}-{P_{\lambda }}\in W^{d-1,d}({\mathbb {R}}^N;{\mathbb {R}}^N)$ owing to Definition 6.1, the identity $\tau _h\left( \frac{\partial ^{a}}{\partial x^{a}}{P_{\lambda }}\right) =\frac{\partial ^{a}}{\partial x^{a}}\left( \tau _h{P_{\lambda }}\right)$, as well as Definition 6.1, Assertion 2.

In particular, choosing $\bar{\lambda }:=(1,\dots ,1)$ we have

$$\begin{aligned}&\sup _{u\in {\mathcal {F}}}\left\{ \left\| \tau _h({v}^n_u) -{v}^n_u\right\| _{L^1({2}Q;{{{\mathbb {R}}}^N})}\right\} \\&\quad \le C_\mathscr {A}\Big (1+\frac{1}{n}\Big ) \sum _{\left|a\right| \le d-1} \left\| \tau _h\left( \frac{\partial ^{a}}{\partial x^{a}} P_{\bar{\lambda }}\right) -\frac{\partial ^{a}}{\partial x^{a}} P_{\bar{\lambda }}\right\| _{L^1({2}Q;{{{\mathbb {R}}}^N})}, \end{aligned}$$

and, in view of (6.2) and Lemma 6.8, we conclude that

$$\begin{aligned}&\lim _{\left|h\right|\rightarrow 0}\sup _{u\in {\mathcal {F}}} \left\{ \left\| \tau _h(v^n_u )-v^n_u \right\| _{L^1({2}Q;{{{\mathbb {R}}}^N})}\right\} \\&\le C_\mathscr {A}\Big (1+\frac{1}{n}\Big ) \lim _{\left|h\right|\rightarrow 0} \sum _{\left|a\right|= d-1} \left\| \tau _h \Big (\frac{\partial ^{a}}{\partial x^{a}}P_{\bar{\lambda }}\Big ) -\frac{\partial ^{a}}{\partial x^{a}}P_{\bar{\lambda }}\right\| _{L^1({2}Q;{{{\mathbb {R}}}^N})}=0 \end{aligned}$$

for every $n\in {{\mathbb {N}}}$, which yields (6.9).

By (6.8), for $n\in {{\mathbb {N}}}$ fixed, for every $h\in {\mathbb {R}}^N$ with $|h|<1$, and for every $u\in {{\mathcal {F}}}$ there holds

$$\begin{aligned} \left\| \tau _h u-u\right\| _{L^1\left( \frac{3}{2}Q;{{{\mathbb {R}}}^N}\right) }&\le \left\| \tau _h u-\tau _h v^n_u\right\| _{L^1\left( \frac{3}{2}Q;{{{\mathbb {R}}}^N}\right) }\\&\quad +\left\| \tau _h v^n_u-v^n_u\right\| _{L^1(\frac{3}{2}Q;{{{\mathbb {R}}}^N})}+\left\| v^n_u-u\right\| _{L^1 \left( \frac{3}{2}Q;{{{\mathbb {R}}}^N}\right) }\\&\le 2\left\| v^n_u-u\right\| _{L^1(2Q;{{{\mathbb {R}}}^N})} +\left\| \tau _h v^n_u-v^n_u\right\| _{L^1(2Q;{{{\mathbb {R}}}^N})} \end{aligned}$$

The thesis follows then by (6.8), (6.9), and Proposition 6.6. $\square$

We close this subsection by proving a compactness and lower-semicontinuity result for functions with uniformly bounded $BV_{{\mathscr {B}}_n}$ norms. We recall that the definition of $M_\mathscr {A}$ is found in (6.2).

#### Proposition 6.10

Let $\left\{ {{\mathscr {B}}_n}\right\} _{n=1}^\infty \subset \Pi _{\mathscr {A}}$ be such that ${\mathscr {B}}_n\rightarrow {\mathscr {B}}$ in $\ell ^{\infty }$ and the constants $C_{{\mathscr {B}}_n}$ in (6.5) are uniformly bounded. For every $n\in {\mathbb {N}}$ let $v_n\in BV_{{\mathscr {B}}_n}(Q;{\mathbb {R}}^N)$ be such that

6.11 $$\begin{aligned} \sup \left\{ \left\| v_n\right\| _{BV_{{\mathscr {B}}_n}(Q;{\mathbb {R}}^N)}:\,\,n\in {\mathbb {N}}\right\} <+\infty . \end{aligned}$$

Then there exists $v\in BV_{{\mathscr {B}}}(Q;{\mathbb {R}}^N)$ such that, up to a subsequence (not relabeled),

6.12 $$\begin{aligned} v_n\rightarrow v\text { strongly in }L^1(Q;{\mathbb {R}}^N), \end{aligned}$$

and

6.13 $$\begin{aligned} {{\mathscr {B}}_nv_n}\mathrel {\mathop {\rightharpoonup }\limits ^{*}}{{\mathscr {B}}v}\ \text {weakly}^{*}\ \text {in }{\mathcal {M}_b}(Q;{{\mathbb {R}}^{N\times N}}). \end{aligned}$$

#### Proof

Let ${v_n}$ satisfy (6.11). With a slight abuse of notation we still indicate by $v_n$ the ${BV_{{\mathscr {B}}_n}}$ continuous extension of the above maps to ${\mathbb {R}}^N$ (see Lemma 6.3). Let $\phi \in C^{\infty }_c(2Q;{\mathbb {R}}^N)$ be a cut-off function such that $\phi \equiv 1$ on *Q*, and for every $n\in {\mathbb {N}}$ let $\tilde{v}_n$ be the map $\tilde{v}_n:=\phi v_n$. Note that $\textrm{supp}\, \tilde{v}_n\subset \subset 2Q$. Additionally, by Lemma 6.3 there holds

6.14 $$\begin{aligned} \left\| \tilde{v}_n\right\| _{BV_{{\mathscr {B}}_n}(2Q;{{{\mathbb {R}}}^N})}&\le \left\| v_n\right\| _{L^1(2Q;{{{\mathbb {R}}}^N})}{+} \left|{\mathscr {B}}_n v_n\right|_{M_b(2Q;{\mathbb {R}}^{N\times N})}\nonumber \\&\quad +\left\| \sum _{i=1}^N {B^i_n} \frac{\partial \phi }{\partial x_i}\right\| _{L^{\infty } (2Q;{\mathbb {M}}^{N^3})}\left\| v_n\right\| _{L^1(2Q;{\mathbb {R}}^N)}\nonumber \\&\le C_1\left\| {v}_n\right\| _{BV_{{\mathscr {B}}_n}(2Q;{{{\mathbb {R}}}^N})} \le C_2 \left\| {v}_n\right\| _{BV_{{\mathscr {B}}_n}(Q;{{{\mathbb {R}}}^N})}, \end{aligned}$$

where in the last inequality we used Lemma 6.3, and where the constants $C_1$ and $C_2$ depend only on the cut-off function $\phi$. To prove (6.12) we first show that

6.15 $$\begin{aligned} \lim _{\left|h\right|\rightarrow 0}\sup _{n\in {\mathbb {N}}}\left\{ \left\| \tau _h \tilde{v}_n -\tilde{v}_n\right\| _{L^1({{{\mathbb {R}}}^N};{{{\mathbb {R}}}^N})}\right\} =0, \end{aligned}$$

where we recall $\tau _h$ from Theorem 6.6. Arguing as in the proof of (6.10), by (6.14) we deduce that for |*h*| small enough, since $\textrm{supp}\,\phi \subset \subset 2Q$,

$$\begin{aligned}&\left\| \tau _h \tilde{v}_n-\tilde{v}_n\right\| _{L^1({{{\mathbb {R}}}^N};{{{\mathbb {R}}}^N})} =\left\| \tau _h \tilde{v}_n-\tilde{v}_n\right\| _{L^1(2Q;{{{\mathbb {R}}}^N})}\\&\quad \le C\left[ \sum _{\left|a\right|\le d-1} \left\| \tau _h \left( \frac{\partial ^{a}}{\partial x^{a}}{P_{\lambda }}\right) -\frac{\partial ^{a}}{\partial x^{a}}{P_{\lambda }}\right\| _{L^1(2Q;{{{\mathbb {R}}}^N})}\right] \left|{\mathscr {B}}\tilde{v}_n\right|_{{\mathcal {M}}_b(2Q;{{\mathbb {R}}^{N\times N}})}\\&\quad \le C\left[ \sum _{\left|a\right|\le d-1} \left\| \tau _h \left( \frac{\partial ^{a}}{\partial x^{a}}{P_{\lambda }}\right) -\frac{\partial ^{a}}{\partial x^{a}}{P_{\lambda }}\right\| _{L^1(2Q;{{{\mathbb {R}}}^N})}\right] \left\| v_n\right\| _{BV_{{\mathscr {B}}}(Q;{{\mathbb {R}}^{N\times N}})} \end{aligned}$$

for every $n\in {\mathbb {N}}$. Property (6.15) follows by (6.2). Owing to Proposition 6.6, we deduce (6.12).

We now prove (6.13). Let $\varphi \in C_c^\infty (Q;{{\mathbb {R}}^{N\times N}})$ be such that $\left|\varphi \right|\le 1$. Then

$$\begin{aligned} \lim _{n\rightarrow \infty }\int _Q\varphi \cdot d({\mathscr {B}}_nv_n)&= \lim _{n\rightarrow \infty }\sum _{i,j=1}^N \int _Q \varphi _{ij}d\left( \sum _{k,l=1}^N (B_n)_{ijl}^k\frac{\partial (v_n)_l}{\partial x_k}\right) \\&= \lim _{n\rightarrow \infty }\sum _{i,j,k,l=1}^N \int _Q \varphi _{ij}d\left( (B_n)_{ijl}^k\frac{\partial (v_n)_l}{\partial x_k}\right) \\&=- \lim _{n\rightarrow \infty }\sum _{i,j,k,l=1}^N \int _Q (v_n)_{l}{ (B_n)_{ijl}^k\frac{\partial \varphi _{ij}}{\partial x_k}}dx\\&=- \sum _{i,j,k,l=1}^N \int _Q v_{l}{ (B)_{ijl}^k\frac{\partial \varphi _{ij}}{\partial x_k}}dx \end{aligned}$$

where in the last step we used the fact that $v_n\rightarrow v$ strongly in $L^1(Q)$ and ${\mathscr {B}}_n\rightarrow {\mathscr {B}}$ in $\ell ^{\infty }$.

This completes the proof of (6.13) and of the proposition. $\square$

#### Proof of Theorem 6.5

Let ${\mathscr {B}}\in \Pi _\mathscr {A}$ be given. The fact that ${\mathscr {B}}$ satisfies Assumption 3.2 follows by Propositions 6.7 and 6.9. The fulfillment of Assumption 3.3 is a direct consequence of Proposition 6.10. $\square$

### Training Scheme with Fixed and Multiple Operators $\mathscr {A}$

In this subsection we provide a construction of training sets associated to a given differential operator $\mathscr {A}$, namely collection of differential operators ${\mathscr {B}}$ for which our training scheme is well-posed (see Definitions 5.1 and 5.2). We first introduce a collection $\Sigma [\mathscr {A}]$ for a given operator $\mathscr {A}$ of order $d\in {\mathbb {N}}$.

#### Definition 6.11

Let $\mathscr {A}$ be a differential operator of order $d\in {\mathbb {N}}$. We denote by $\hat{\Sigma }[\mathscr {A}]$ the set

$$\begin{aligned} \hat{\Sigma }[\mathscr {A}]:=\left\{ {\mathscr {B}}\in \Pi _\mathscr {A}: \left\| {\mathscr {B}}\right\| _{\ell ^{\infty }}\le 1\right\} . \end{aligned}$$

The first result of this subsection is the following.

#### Theorem 6.12

Let $\mathscr {A}$ be a differential operator of order $d\in {\mathbb {N}}$, and assume that $\Sigma [\mathscr {A}]$ is a non-empty subset of $\hat{\Sigma }[\mathscr {A}]$ which is closed in the $\ell ^\infty$ convergence with respect of the property of having finite-dimensional null space. Then the collection $\Sigma [\mathscr {A}]$ is a training set (see Definition 5.1).

#### Proof

By the definition of $\Sigma [\mathscr {A}]$ we just need to show that $\Sigma [\mathscr {A}]$ is closed in $\ell ^{\infty }$. Let $u\in C^\infty (Q;{\mathbb {R}}^N)$ and $\left\{ {{\mathscr {B}}_n}\right\} _{n=1}^\infty \subset {\Sigma [\mathscr {A}]}$ be given. Then, up to a subsequence (not relabeled), we may assume that ${\mathscr {B}}_n\rightarrow {\mathscr {B}}$ in $\ell ^{\infty }$. We claim that ${\mathscr {B}}\in \Pi _\mathscr {A}$.

The fact that ${\mathcal {N}}({\mathscr {B}})$ is finite-dimensional follows by definition. To conclude the proof of the theorem we still need to show that $(\mathscr {A},{\mathscr {B}})$ satisfies Definition 6.1, Assertion 2. Let *U* be an open set in ${\mathbb {R}}^N$ such that $Q\subset U$. Let $u\in C^{\infty }_c(U;{{\mathbb {R}}^{N\times N}})$ and let $v\in BV_{{\mathscr {B}}}(U;{{{\mathbb {R}}}^N})$. By Proposition 6.7 there exists $\left\{ {v_k}\right\} _{k=1}^\infty \subset C^\infty (U;{{{\mathbb {R}}}^N})$ such that

6.16 $$\begin{aligned} v_k\rightarrow v\text { strongly in }L^1(U;{{{\mathbb {R}}}^N})\text { and } \left|{\mathscr {B}}v_k\right|_{{\mathcal {M}}_b(U;{\mathbb {R}}^{N\times N})} \rightarrow \left|{\mathscr {B}}v\right|_{{\mathcal {M}}_b(U;{\mathbb {R}}^{N\times N})}. \end{aligned}$$

Integrating by parts we obtain

$$\begin{aligned}&\left\| \left( {\mathscr {A}}u\right) _i*(v_k)_i\right\| _{L^1(U;{{{\mathbb {R}}}^N})} \le C_{\mathscr {A}}\left[ \sum _{\left|a\right|\le d-1} \left\| \frac{\partial ^{a}}{\partial x^{a}}u\right\| _{L^1(U;{{{\mathbb {R}}}^N})}\right] \left|{\mathscr {B}}_n v_k\right|_{{\mathcal {M}}_b(U;{{\mathbb {R}}^{N\times N}})}, \end{aligned}$$

for every $i=1,\ldots , N$. Taking the limit as $n\rightarrow \infty$ first, and then as $k\rightarrow \infty$, since ${\mathscr {B}}_n\rightarrow {\mathscr {B}}$ in $\ell ^{\infty }$ and in view of (6.16), we conclude that

$$\begin{aligned}&\left\| \left( {\mathscr {A}}u\right) _i*(v_k)_i\right\| _{L^1(U;{{{\mathbb {R}}}^N})} \le C_{\mathscr {A}}\left[ \sum _{\left|a\right|\le d-1} \left\| \frac{\partial ^{a}}{\partial x^{a}}u\right\| _{L^1(U;{{{\mathbb {R}}}^N})}\right] \left|{\mathscr {B}}v\right|_{{\mathcal {M}}_b(U;{{\mathbb {R}}^{N\times N}})}. \end{aligned}$$

The proof of the second part of Assertion 2 is analogous. This shows that $(\mathscr {A},{\mathscr {B}})$ satisfies Definition 6.1 and concludes the proof of the theorem.

#### Remark 6.13

We note that the result of Theorem 6.12 still holds if we replace the upper bound 1 in Definition 6.11 with an arbitrary positive constant.

We additionally point out that requiring that the finite-dimensional-kernel property is preserved in the limit passage automatically ensures the existence of a lower bound on the $\ell ^\infty$-norms of the operators. In other words, the null operator is not included in our analysis.

As a final remark, we stress that, if $\hat{\Sigma }[\mathscr {A}]$ contains an operator $\bar{{\mathscr {B}}}$ with finite-dimensional null space, then a training set $\Sigma [\mathscr {A}]\subset \hat{\Sigma }[\mathscr {A}]$ being closed in the $\ell ^\infty$-norm with respect to the property of having finite-dimensional null space can be constructed by taking the intersection of $\hat{\Sigma }[\mathscr {A}]$ with a small enough neighborhood of $\bar{{\mathscr {B}}}$ in the $\ell ^\infty$-topology.

In fact, denoting by $B^i\in {\mathbb {M}}^{N^3}$, $i=1,\dots ,N$, the coefficients of $\bar{{\mathscr {B}}}$, the symbol of $\bar{{\mathscr {B}}}$ is defined as

$$\begin{aligned} {{\mathbb {B}}}[\xi ]:=\sum _{i=1}^N \xi _i B^i\quad \text {for every } \xi =(\xi _1,\dots ,\xi _N)\in {\mathbb {C}}^{N}\setminus \{0\}. \end{aligned}$$

The condition of having finite-dimensional null space is equivalent to the so-called ${\mathbb {C}}$-ellipticity condition, which consists in the injectivity of the map ${\mathbb {B}}[\xi ]$ as a linear map on ${\mathbb {C}}^{N}\setminus \{0\}$ for every $\xi \in {\mathbb {C}}^{N}{\setminus } \{0\}$ [see Breit et al. (2017, Section 2.3)]. By linearity, this, in turn, can be reduced to the condition of the map ${\mathbb {B}}[\xi ]$ being injective on ${\mathbb {C}}^{N}\setminus \{0\}$ for every $\xi$ in $B_{{\mathbb {C}}}(0,1)\setminus \{0\}$, where $B_{{\mathbb {C}}}(0,1)$ is the unit ball centered in the origin in the complex plane. In particular, it is a stable condition with respect to small $\ell ^\infty$-perturbations of the coefficients.

We now consider the case of multiple operators $\mathscr {A}$.

#### Definition 6.14

We say that collection ${\mathcal {A}}$ of differential operators $\mathscr {A}$ is a training set builder if

6.17 $$\begin{aligned} \sup \left\{ C_\mathscr {A}:\,\, \mathscr {A}\in {\mathcal {A}}\right\} <+\infty \text { and }\lim _{\left|h\right|\rightarrow 0} \sup \left\{ M_\mathscr {A}(h): \,\, \mathscr {A}\in {\mathcal {A}}\right\} =0, \end{aligned}$$

where $C_\mathscr {A}$ and $M_\mathscr {A}(h)$ are defined in (6.1) and (7.5), respectively.

We then define the class $\Sigma [{\mathcal {A}}]$ via

$$\begin{aligned} {\Sigma [{\mathcal {A}}]:=\textrm{convex}\,\,\textrm{hull} \left( \bigcup _{\mathscr {A}\in {\mathcal {A}}} \Sigma [\mathscr {A}]\right) }, \end{aligned}$$

where for every $\mathscr {A}\in {\mathcal {A}}$, $\Sigma [\mathscr {A}]$ is the class defined in Definition 6.11.

We close this section by proving the following theorem.

#### Theorem 6.15

Let ${\mathcal {A}}$ be a training set builder. Then $\Sigma [{\mathcal {A}}]$ is a training set.

#### Proof

The proof of this theorem follows the argument in the proof of Theorem 6.12 using the fact that the two critical constants $M_\mathscr {A}(h)$ and $C_\mathscr {A}$, in (6.2) and (6.1), respectively, are uniformly bounded due to (6.17). $\square$

## Explicit Examples and Numerical Observations

In this section we exhibit several explicit examples of operators $\mathscr {A}$ and training sets ${\Sigma [\mathscr {A}]}$, we provide numerical simulations and we make some observations derived from them.

### The Existence of Fundamental Solutions of Operators $\mathscr {A}$

One important requirement in Definition 6.1 is the existence of the fundamental solution ${P_{\lambda }}\in L^1({{{\mathbb {R}}}^N},{{{\mathbb {R}}}^N})$ of a given operator $\mathscr {A}$. A result in this direction can be found in Hsiao and Wendland (2008, p. 351, Section 6.3), where an explicit form of the fundamental solution for Agmon-Douglis-Nirenberg elliptic systems with constant coefficients is provided.

#### Remark 7.1

In the case in which $N=2$, $\mathscr {A}$ has order 2 and satisfies the assumptions in Hsiao and Wendland (2008, p. 351, Section 6.3), the fundamental solution ${P_{\lambda }}$ can be written as

7.1 $$\begin{aligned} {P_{\lambda }}(x,y)=\frac{1}{8\pi ^2}(\Delta L_y) \int _{\left|\eta \right|=1, \eta \in {\mathbb {R}}^2}\left( (x-y)\cdot \eta \right) ^2 \log \left| (x-y)\cdot \eta \right| R_\mathscr {A}d\omega _\eta , \end{aligned}$$

where *L* denotes the fundamental solution of Laplace’s equation, $R_\mathscr {A}$ denotes a constant depending on $\mathscr {A}$, and the integration is taken over the unit circle $\left|\eta \right| = 1$ with arc length element $d\omega _\eta$.

In the special case in which

7.2 $$\begin{aligned} \mathscr {A}w:=\Delta w+\nabla ({\textrm{div}}w) \quad \text { for}\ w\in {\mathcal {D}}'(Q;{{{\mathbb {R}}}^{2}}), \end{aligned}$$

the fundamental solution $P_{\alpha }$, with $\mathscr {A}P_{\alpha }=\alpha \delta$ for $\alpha \in {\mathbb {R}}^2$, is given by

$$\begin{aligned} P_{\alpha }(x):=\frac{3\alpha }{8\pi }\log \frac{1}{\left|x\right|} +\frac{x}{8\pi }\frac{\alpha \cdot \left|x\right|}{\left|x\right|^2}. \end{aligned}$$

We observe that $\nabla P_{\alpha }$ is positively homogeneous of degree $-1(= 1-N)$. Also, since $R_\mathscr {A}$ in (7.1) is a constant, $\nabla {P_{\lambda }}$ must have the same homogeneity as $\nabla P_{\alpha }$, which is $1-N$.

#### Proposition 7.2

Let $\mathscr {A}$ be a differential operator of order $d\in {\mathbb {N}}$, and assume that its fundamental solution ${P_{\lambda }}$ is such that $\frac{\partial ^{a}}{\partial x^{a}}{P_{\lambda }}$ is positively homogeneous of degree $1-N$ for all multi-indexes $a\in {\mathbb {N}}^N$ with $\left|a\right|=d-1$. Then property (6.2) is satisfied.

#### Proof

Let $s\in (0,1)$ be fixed. Since $\frac{\partial ^{a}}{\partial x^{a}}P_\lambda$ is positively homogeneous of degree $1-N$ for all multi-indexes $a\in {\mathbb {N}}^N$ with $\left|a\right|=d-1$, by Temam (1983, Lemma 1.4) we deduce the estimate

7.3 $$\begin{aligned}&\sum _{\left|a\right|=d-1}\left|\tau _h\left( \frac{\partial ^{a}}{\partial x^{a}} {P_{\lambda }}(x)\right) -\frac{\partial ^{a}}{\partial x^{a}} {P_{\lambda }}(x)\right|\nonumber \\&\quad \le C\left[ \max \left\{ \sup \left\{ \left|\nabla ^{d-1} {P_{\lambda }}(z)\right|: \,\, {\left|z\right|=1}\right\} ,\,\, \sup \left\{ \left|\nabla ^d{P_{\lambda }}(z)\right|: \,\, {\left|z\right|=1}\right\} \right\} \right] \nonumber \\&\qquad \cdot \left|h\right|^{s}\left[ \frac{1}{\left|x\right|^{N-1+s}} +\frac{1}{\left|x+h\right|^{N-1+s}}\right] . \end{aligned}$$

for every $x\in {\mathbb {R}}^N$, $0\le s\le 1$, and $\left|h\right|\le 1/2$, where the constant *C* is independent of *x* and *h*.

Next, for every bounded open set $U\subset {\mathbb {R}}^N$ satisfying $Q\subset U$ we have

7.4 $$\begin{aligned}&\int _U\frac{1}{\left|x\right|^{N-1+s}}dx\le \int _{B(0,2)} \frac{1}{\left|x\right|^{N-1+s}}dx+\int _{U\setminus B(0,2)} \frac{1}{\left|x\right|^{N-1+s}}dx\nonumber \\&\quad \le 2\pi \int _0^2 r^{-s}dr+\frac{1}{2^{N-1+s}}| U\setminus B(0,2)|<+\infty , \end{aligned}$$

The analogous computation holds for $\frac{1}{\left|x+h\right|^{N-1+s}}$. Since $P_\lambda$ is a fundamental solution and $\mathscr {A}P_\lambda =\lambda \delta$, we have that $P_\lambda \in C^\infty ({{{\mathbb {R}}}^N}{\setminus } B(0,\varepsilon ))$ for every $\varepsilon >0$. In particular,

7.5 $$\begin{aligned} \max \left\{ \sup \left\{ \left|\nabla ^{d-1} P(z)\right|: \,\, {\left|z\right|=1}\right\} ,\,\, \sup \left\{ \left|\nabla ^dP(z)\right|: \,\, {\left|z\right|=1}\right\} \right\} =:M <+\infty . \end{aligned}$$

This, together with (7.3) and (7.4), yields

$$\begin{aligned} \left\| \sum _{\left|a\right|=d-1}\left|\tau _h\left( \frac{\partial ^{a}}{\partial x^{a}} P_\lambda (x)\right) -\frac{\partial ^{a}}{\partial x^{a}} P_\lambda (x)\right|\right\| _{L^1(U;{\mathbb {R}}^N)}\le C M\left|h\right|^s , \end{aligned}$$

for some $C>0$, and thus

$$\begin{aligned} \lim _{h\rightarrow \infty }\left\| \sum _{\left|a\right|=d-1}\left|\tau _h \left( \frac{\partial ^{a}}{\partial x^{a}} P_\lambda (x)\right) -\frac{\partial ^{a}}{\partial x^{a}} P_\lambda (x)\right|\right\| _{L^1(U;{\mathbb {R}}^N)}=0, \end{aligned}$$

and (6.2) is established. $\square$

#### Remark 7.3

As a corollary of Proposition 7.2 and Remark 7.1, we deduce that all operators $\mathscr {A}$ satisfying the assumptions in Hsiao and Wendland (2008, p. 351, Section 6.3) comply with Definition 6.1, Assertion 1. In particular, differential operators $\mathscr {A}$ which can be written in the form $\mathscr {A}={\mathscr {B}}^*\circ \mathscr {C}$, where ${\mathscr {B}}^*$ is the first order differential operator associated to ${\mathscr {B}}$ and having as coefficients the transpose of the matrices $B^i$, $i=1,\dots ,N$, and where $\mathscr {C}$ is a differential operator of order $d-1$ having constant coefficients, are such that $(\mathscr {A},{\mathscr {B}})$ complies with Definition 6.1.

### The Unified Approach to $TGV^2$ and $NsTGV^2$: An Example of $\Sigma [\mathscr {A}]$

In this section we give an explicit construction of an operator $\mathscr {A}$ such that the seminorms $NsTGV^2$ and $TGV^2$, as well as a continuum of topologically equivalent seminorms connecting them, can be constructed as operators ${\mathscr {B}}\in \Sigma [\mathscr {A}]$.

We start by recalling the definition of the classical symmetrized gradient,

7.6 $$\begin{aligned} {\mathcal {E}} v = \frac{\nabla v+(\nabla v)^T}{2}=\begin{bmatrix} \partial _1 v_1 &{}\frac{(\partial _1 v_2+\partial _2 v_1)}{2}\\ \frac{(\partial _1 v_2+\partial _2 v_1)}{2} &{} \partial _2 v_2 \end{bmatrix}, \end{aligned}$$

for $$v=(v_1,v_2)\in C^\infty (Q;{\mathbb {R}}^2)$$. Let

$$\begin{aligned} B^1_{\textrm{sym}}=\left[ \begin{array}{cc|cc} 1 &{} \ 0 &{} \ 1/2 &{} \ 0\\ 0&{} \ 1/2 &{} \ 0&{} \ 0\\ \end{array}\right] \text { and } B^2_{\textrm{sym}}=\left[ \begin{array}{cc|cc} 0 &{} \ 0 &{} \ 1/2 &{} \ 0\\ 0&{} \ 1/2 &{} \ 0 &{} \ 1 \\ \end{array}\right] , \end{aligned}$$

and let ${\mathscr {B}}_{\textrm{sym}}(v)$ be defined as in (2.1) with $B^1_{\textrm{sym}}$ and $B^2_{\textrm{sym}}$ as above. Then ${\mathscr {B}}_{\textrm{sym}} (v) ={\mathcal {E}} v$ for all $v\in C^\infty (Q;{{{\mathbb {R}}}^{2}})$, and ${\mathcal {N}}({\mathscr {B}}_{\textrm{sym}})$ is finite dimensional. In particular,

$$\begin{aligned} {\mathcal {N}}({\mathscr {B}}_{\textrm{sym}})=\left\{ v(x)=\alpha \Big (\begin{array}{c} x_2\\ -x_1 \end{array}\Big )+b:\,\alpha \in {{\mathbb {R}}} \quad \text {and}\quad b\in {{\mathbb {R}}}^2\right\} . \end{aligned}$$

The first part of Definition 6.1 follows from Remark 7.3. Next we verify that (6.1) holds. Indeed, choosing $\mathscr {A}$ as in (7.2), we first observe that

7.7 $$\begin{aligned} (\mathscr {A}w)*v&= \sum _{j=1}^N\left[ \Delta w_j+\partial _j {\textrm{div}}(w)\right] *v_j =\sum _{i,j=1}^N (\partial _i w_j+\partial _j w_i)*\partial _i v_j\nonumber \\&={\frac{1}{2}} \sum _{i,j=1}^N(\partial _i w_j+\partial _j w_i) *(\partial _i v_j+\partial _j v_i) ={\frac{1}{2}} {({\mathscr {B}}_{\textrm{sym}} w)*({\mathscr {B}}_{\textrm{sym}}v)}, \end{aligned}$$

for every $w\in W^{1,2}(Q;{{{\mathbb {R}}}^{2}})$ and $v\in C^\infty _c(Q;{{{\mathbb {R}}}^{2}})$. That is, for every open set $U\subset {\mathbb {R}}^N$ such that $Q\subset U$ we have

$$\begin{aligned} \left|(\mathscr {A}w)*v\right|_{{\mathcal {M}_b}(U;{\mathbb {R}}^2)}&\le {\frac{1}{2}} \left|({\mathscr {B}}_{\textrm{sym}} w)*({\mathscr {B}}_{\textrm{sym}}v)\right|_{{\mathcal {M}_b}(U;{{\mathbb {M}}}^{2\times 2})}\\&\le {\frac{1}{2}} \left\| \nabla w\right\| _{L^1(U;{{\mathbb {M}}^{2\times 2}})} \left|{\mathscr {B}}_{\textrm{sym}} (v)\right|_{{\mathcal {M}_b}(U;{{\mathbb {M}}}^{2\times 2})}. \end{aligned}$$

The same computation holds for $w\in C^{\infty }_c(Q;{{{\mathbb {R}}}^{2}})$ and $v\in BV_{{\mathscr {B}}}(Q;{{{\mathbb {R}}}^{2}})$. This proves that Assertion 2 in Definition 6.1 is also satisfied.

We finally construct an example of a training set $\Sigma [\mathscr {A}]$. For every $0\le s,t\le 1$, we define

$$\begin{aligned} B_t:=\left[ \begin{array}{cc|cc} 1 &{}\ 0 &{} \ t &{} \ 0\\ 0&{} \ (1-t) &{} \ 0&{} \ 0\\ \end{array}\right] \text { and } B_s:=\left[ \begin{array}{cc|cc} 1 &{} \ 0 &{} \ s &{} \ 0\\ 0&{} \ 1-s &{} \ 0 &{} \ 0 \\ \end{array}\right] , \end{aligned}$$

and we set

7.8 $$\begin{aligned} {\mathscr {B}}_{s,t}( v): = B_t \partial _1 v+B_s\partial _2 v=\begin{bmatrix} \partial _1 v_1 &{}(1-t)\partial _1 v_2+(1-s)\partial _2 v_1\\ t\partial _1 v_2+s\partial _2 v_1 &{} \partial _2 v_2 \end{bmatrix}. \end{aligned}$$

By a straightforward computation, we obtain that ${\mathcal {N}}({\mathscr {B}}_{s,t})$ is finite dimensional for every $0\le s,t\le 1$. Additionally, Assertion 1 in Definition 6.1 follows by adapting the arguments in Remark 7.3. Finally, arguing exactly as in (7.7), we obtain that

$$\begin{aligned} (\mathscr {A}w)*v = ({\mathscr {B}}_{t,s} w)*({\mathscr {B}}_{s,t} (v)), \text { for every }w,v\in C^\infty (\bar{Q};{{{\mathbb {R}}}^{2}}), \end{aligned}$$

which implies that

$$\begin{aligned} \left|(\mathscr {A}w)*v \right|_{{\mathcal {M}_b}(Q;{\mathbb {R}}^2)}&\le \left\| {\mathscr {B}}_{t,s} w\right\| _{L^1(Q;{\mathbb {M}}^{2\times 2})} \left|{\mathscr {B}}_{s,t} (v)\right|_{{\mathcal {M}_b}(Q;{{\mathbb {M}}^{2\times 2}})}\\&\le 2\left\| \nabla w\right\| _{L^1(Q;{{\mathbb {R}}^{N\times N}})}\left|{\mathscr {B}}_{s,t} (v)\right|_{{\mathcal {M}_b}(Q;{{\mathbb {M}}^{2\times 2}})}. \end{aligned}$$

Hence, we deduce again Statement 2 in Definition 6.1. Therefore, the collection $\Sigma [\mathscr {A}]$ given by

$$\begin{aligned} \Sigma [\mathscr {A}]:=\left\{ {\mathscr {B}}_{s,t}:\,\, 0\le s,t\le 1\right\} \end{aligned}$$

is a training set according to Definition 6.11. We remark that $\Sigma [\mathscr {A}]$ includes the operator $TGV^2$ (with $s=t=1/2$) and the operator $NsTGV^2$ (with $t=0$ and $s=1$), as well as a collection of all “interpolating” regularizers. In other words, our training scheme $({\mathcal {T}}^2_{\theta })$ with training set $\Sigma [\mathscr {A}]$ is able to search for optimal results in a class of operators including the commonly used $TGV^2$ and $NsTGV^2$, as well as any interpolation regularizer.

#### Comparison with Other Works

In Brinkmann et al. (2019) the authors analyze a range of first order linear operators generated by diagonal matrixes. To be precise, letting ${B}=\textrm{diag}(\beta _1,\beta _2,\beta _3,\beta _4)$, Brinkmann et al. (2019) treats first order operators ${\mathscr {B}}$ defined as

$$\begin{aligned} {\mathscr {B}}v := Q \cdot B \cdot Q\cdot (\nabla v)^T, \end{aligned}$$

where

$$\begin{aligned} Q:=\begin{bmatrix} 0 &{} \quad 1&{} \quad -1&{} \quad 0\\ 1 &{} \quad 0 &{} \quad 0 &{} \quad 1\\ -1&{} \quad 0&{} \quad 0&{} \quad 1\\ 0 &{} \quad 1&{} \quad 1&{} \quad 0 \end{bmatrix} \text { and } \nabla v = [\partial _1 v_1, \partial _1 v_2, \partial _2v_1,\partial _2v_2]. \end{aligned}$$

That is, instead of viewing $\nabla v$ as a $2\times 2$ matrix as we do, in Brinkmann et al. (2019) $\nabla v$ is represented as a vector in ${\mathbb {R}}^4$. In this way, the symmetric gradient ${\mathcal {E}}v$ in (7.6) can be written as

$$\begin{aligned} {\mathcal {E}}v&= Q\cdot \textrm{diag}(0,1/2,1/2,1/2) \cdot Q \cdot (\nabla v)^T = \begin{bmatrix} 1 &{} \quad 0&{} \quad 0&{} \quad 0\\ 0 &{} \quad 1/2 &{} \quad 1/2 &{} \quad 0\\ 0&{} \quad 1/2&{} \quad 1/2&{} \quad 0\\ 0 &{} \quad 0 &{} \quad 0&{} \quad 1 \end{bmatrix} \cdot [\partial _1 v_1, \partial _1 v_2, \partial _2v_1,\partial _2v_2]^T\\&= [\partial _1 v_1, 0.5(\partial _1 v_2+\partial _2 v_1),0.5 (\partial _1 v_2+\partial _2 v_1),\partial _2v_2]. \end{aligned}$$

However, the representation above does not allow to consider skewed symmetric gradients ${\mathscr {B}}_{s,t}(v)$ with the structure introduced in (7.8). Indeed, let $s=t=0.2$. We have

$$\begin{aligned} {\mathscr {B}}_{0.2,0.2}(v)=\begin{bmatrix} \partial _1 v_1 &{}0.8\partial _1 v_2+0.8\partial _2 v_1\\ 0.2\partial _1 v_2+0.2\partial _2 v_1 &{} \partial _2 v_2 \end{bmatrix}. \end{aligned}$$

Rewriting the matrix above as a vector in ${\mathbb {R}}^4$, we obtain

$$\begin{aligned} {\mathscr {B}}_{0.2,0.2}(v)&=[\partial _1 v_1, 0.2(\partial _1 v_2 +\partial _2 v_1),0.8(\partial _1 v_2+\partial _2 v_1),\partial _2v_2]\\&=\begin{bmatrix} 1 &{} \quad 0&{} \quad 0&{} \quad 0\\ 0 &{} \quad 0.8 &{} \quad 0.8 &{} \quad 0\\ 0&{} \quad 0.2&{} \quad 0.2&{} \quad 0\\ 0 &{} \quad 0 &{} \quad 0&{} \quad 1 \end{bmatrix} \cdot [\partial _1 v_1, \partial _1 v_2, \partial _2v_1,\partial _2v_2]^T. \end{aligned}$$

That is, we would have

$$\begin{aligned} Q{B'}Q = \begin{bmatrix} 1 &{} \quad 0&{} \quad 0&{} \quad 0\\ 0 &{} \quad 0.8 &{} \quad 0.8 &{} \quad 0\\ 0&{} \quad 0.2&{} \quad 0.2&{} \quad 0\\ 0 &{} \quad 0 &{} \quad 0&{} \quad 1 \end{bmatrix} \text { or } {B'} = \begin{bmatrix} 0 &{} \quad 0&{} \quad 0&{} \quad 0.3\\ 0 &{} \quad 0.5 &{} \quad 0 &{} \quad 0\\ 0&{} \quad 0&{} \quad 0.5&{} \quad 0\\ 0 &{} \quad 0 &{} \quad 0&{} \quad 0.5 \end{bmatrix}, \end{aligned}$$

which are not diagonal matrices. Hence, this example shows that our model indeed covers more operators that those discussed in Brinkmann et al. (2019).

### Numerical Simulations and Observations

Let $\mathscr {A}$ be the operator defined in Sect. 7.2, and let

$$\begin{aligned} \Sigma [\mathscr {A}]:= \left\{ {\mathscr {B}}_{s,t}:\,\,s,t\in [0,1]\right\} \end{aligned}$$

where, for $0\le s,t\le 1$, ${\mathscr {B}}_{s,t}$ are the first order operators introduced in (7.8). As we remarked before, the seminorm $PGV^2_{{\mathscr {B}}_{s,t}}$ interpolates between the $TGV^2$ and $NsTGV^2$ regularizers. We define the cost function ${\mathcal {C}}(\alpha , s,t)$ to be

7.9 $$\begin{aligned} {\mathcal {C}}(\alpha , s,t):=\left\| u_{\alpha ,{\mathscr {B}}_{s,t}}-u_c\right\| _{L^2(Q)}. \end{aligned}$$

From Theorem 5.4 we have that ${\mathcal {C}}(\alpha , s,t)$ admits at least one minimizer $({\tilde{\alpha }},\tilde{s},\tilde{t})\in {\mathbb {R}}^+\times [0,1]\times [0,1]$.

To explore the numerical landscapes of the cost function ${\mathcal {C}}(\alpha ,s,t)$, we consider the discrete box-constraint

7.10 $$\begin{aligned}&(\alpha _0,\alpha _1, s,t)\in \left\{ 0.025,\,0.05,\,0.075,\ldots ,1\right\} \nonumber \\&\quad \times \left\{ 0.025,\,0.05,\,0.075,\ldots , 1\right\} \times \left\{ 0,\,0.025,\,0.05,\,\ldots ,\,1\right\} \times \left\{ 0,\,0.025,\,0.05,\,\ldots ,\,1\right\} . \end{aligned}$$

We perform numerical simulations of the images shown in Fig. : the first image represents a clean image $u_c$, whereas the second one is a noised version $u_\eta$, with heavy artificial Gaussian noise. The reconstructed image $u_{\alpha ,{\mathscr {B}}}$ in Level 2 of our training scheme is computed by using the primal-dual algorithm presented in Chambolle and Pock (2011).

**Fig. 1:**
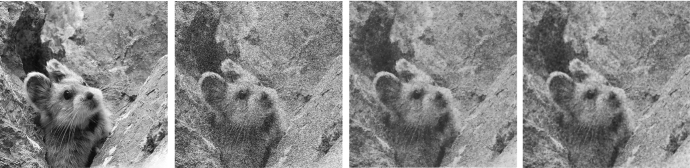
From left to right: the test image of a Pika; a noised version (with heavy artificial Gaussian noise); the optimally reconstructed image with *TGV* regularizer; the optimally reconstructed image with *PGV* regularizer

It turns out that the minimum value of (7.9), taking values in (7.10), is achieved at ${\tilde{\alpha }}_0=0.072$, ${\tilde{\alpha }}_1=0.575$, $\tilde{s}=0.95$, and $\tilde{t}=0.05$. The optimal reconstruction $u_{{\tilde{\alpha }},{\mathscr {B}}_{\tilde{s},\tilde{t}}}$ is the last image in Fig. 1, whereas the optimal result with ${\mathscr {B}}_{s,t}\equiv {\mathcal {E}}$, i.e., $u_{{\tilde{\alpha }},TGV}$, is the third image in Fig. 1. Although the optimal reconstructed image $u_{{\tilde{\alpha }},{\mathscr {B}}_{\tilde{s},\tilde{t}}}$ and $u_{{\tilde{\alpha }},{\mathcal {E}}}$ do not present too many differences to the naked eye, we do have that

$$\begin{aligned} {\mathcal {C}}({\tilde{\alpha }},\tilde{s},\tilde{t})<{\mathcal {C}}({\tilde{\alpha }},0.5,0.5) \end{aligned}$$

(see also Table  below). That is, the reconstructed image $u_{{\tilde{\alpha }},{\mathscr {B}}_{\tilde{s},\tilde{t}}}$ is indeed “better” in the sense of our training scheme ($L^2$-difference).

**Table 1:** Minimum cost value with different regularizers

Regularizer	Optimal solution	Minimum cost value
$TGV^2$	$\tilde{\alpha }_0=0.074$, $\tilde{\alpha }_1=0.625$	${\mathcal {C}}({\tilde{\alpha }},0.5,0.5)=18.653$
$PGV^2$	$\tilde{\alpha }_{0}=0.072$, $\tilde{\alpha }_1=0.575$, $\tilde{s}=0.95$, $\tilde{t}=0.05$	${\mathcal {C}}({\tilde{\alpha }},\tilde{s},\tilde{t})= 17.6478$

The minimum value of the cost function for the $PGV^2$-regularizer is approximately $5\%$ below that of the $TGV^2$-regularizer

To visualize the change of cost function produced by different values of $(s,t)\in [0,1]^2$, we fix $\bar{\alpha }_0=0.072$ and $\bar{\alpha }_1=0.575$ and plot in Fig.  the mesh and contour plot of ${\mathcal {C}}(\bar{\alpha },s,t)$.

**Fig. 2:**
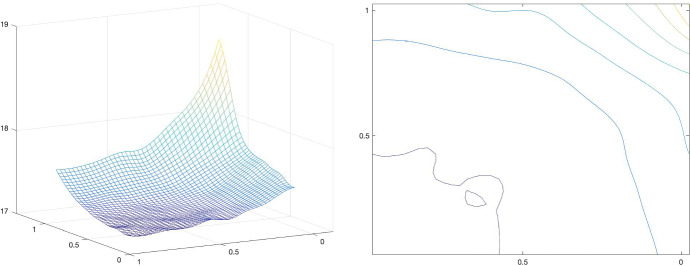
From the left to the right: mesh and contour plot of the cost function ${\mathcal {C}}(\bar{\alpha },s,t)$ in which $\bar{\alpha }=(\bar{\alpha }_0,\bar{\alpha }_1)$ is fixed, $(s,t)\in [0,1]^2$

We again remark that the introduction of $PGV_{\alpha ,{\mathscr {B}}[k]}$ regularizers into the training scheme is only meant to expand the training choices, but not to provide a superior seminorm with respect to the popular choices $TGV^2$ or $NsTGV^2$. The fact whether the optimal regularizer is $TGV^2$, $NsTGV^2$ or an intermediate regularizer is completely dependent on the given training image $u_\eta =u_c+\eta$.

We conclude this section with a further study of the numerical landscapes associated to the cost function ${\mathcal {C}}(\alpha ,s,t)$. We consider also in this second example the discrete box-constraint in (7.10), and we analyze the images shown in Fig. : also in this second example the first image represents the clean image $u_c$, whereas the second one is a noised version $u_\eta$. The reconstructed image $u_{\alpha ,{\mathscr {B}}}$ in Level 2 of our training scheme is again computed by using the primal-dual algorithm presented in Chambolle and Pock (2011).

**Fig. 3:**
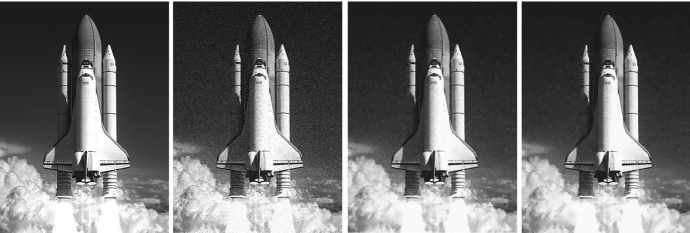
From left to right: the test image of a space shuttle; a noised version (with artificial Gaussian noise); the optimally reconstructed image $u_{{\tilde{\alpha }},{\mathcal {E}}}$, where ${\tilde{\alpha }}_0=5.2$, ${\tilde{\alpha }}_1=1.9$; the optimally reconstructed image $u_{{\tilde{\alpha }},{\mathscr {B}}_{\tilde{s},\tilde{t}}}$, where ${\tilde{\alpha }}_0=5.6$, ${\tilde{\alpha }}_1=1.2$, $\tilde{s}=0.8$, and $\tilde{t}=0.2$

We report that the minimum value of (7.9), taking values in (7.10), is achieved at ${\tilde{\alpha }}_0=5.6$, ${\tilde{\alpha }}_1=1.2$, $\tilde{s}=0.8$, and $\tilde{t}=0.2$. The optimal reconstruction $u_{{\tilde{\alpha }},{\mathscr {B}}_{\tilde{s},\tilde{t}}}$ is the last image in Fig. 3, whereas the optimal result with ${\mathscr {B}}_{s,t}\equiv {\mathcal {E}}$, i.e., $u_{{\tilde{\alpha }},TGV}$, is the third image in Fig. 3. Although the optimal reconstructed image $u_{{\tilde{\alpha }},{\mathscr {B}}_{\tilde{s},\tilde{t}}}$ and $u_{{\tilde{\alpha }},TGV}$ do not present too many differences with respect to our eyesight, we do have, also in this case, that

$$\begin{aligned} \left\| u_{{\tilde{\alpha }},{\mathscr {B}}_{\tilde{s},\tilde{t}}}-u_c\right\| _{L^2(Q)} <\left\| u_{{\tilde{\alpha }},TGV}-u_c\right\| _{L^2(Q)}. \end{aligned}$$

Namely, the reconstructed image $u_{{\tilde{\alpha }},{\mathscr {B}}_{\tilde{s},\tilde{t}}}$ is indeed “better” in the sense of our training scheme ($L^2$-difference).

To visualize the change of cost function produced by different values of $(s,t)\in [0,1]^2$, we fix $\bar{\alpha }_0=5.6$ and $\bar{\alpha }_1=1.9$ and plot in Fig.  the mesh and contour plot of ${\mathcal {C}}(\bar{\alpha },s,t)$ as follows.

**Fig. 4:**
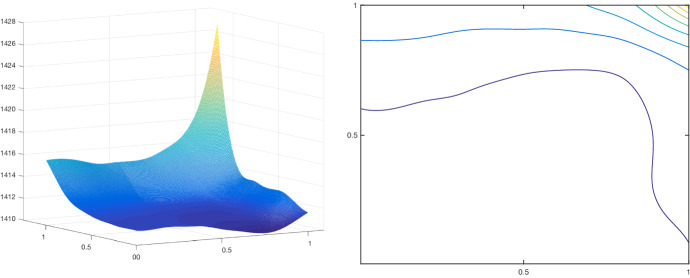
From left to right: mesh and contour plot of the cost function ${\mathcal {C}}(\bar{\alpha },s,t)$ in which $\bar{\alpha }=(\bar{\alpha }_0,\bar{\alpha }_1)$ is fixed, $(s,t)\in [0,1]^2$. The function ${\mathcal {C}}(\bar{\alpha },s,t)$ achieves the minimum at $\tilde{s}=0.8$ and $\tilde{t}=0.2$

## Conclusions

We have introduced a novel class of regularizers providing a generalization of $TGV^2$ to the case in which the higher-order operators can be different from the symmetric gradient. After establishing basic properties of this class of functionals, we have studied well-posedness of a bilevel learning scheme selecting the optimal regularizer in our class in terms of a quadratic cost function. Eventually, we have shown some very first numerical simulations of our scheme. We point out that both examples in Figs. 1 and 3 do not present a clear distinction to the naked eye with respect to their *TGV* counterpart although performing much better in terms of the cost-function landscapes. We conjecture this behavior not to be the general case. Further numerical investigations are beyond the scope of this paper and will be the subject of forthcoming works.

## Data Availability

Data sharing not applicable to this article as no datasets were generated or analysed during the current study.
